# Lower incidence of grade II-IV acute Graft-versus-Host-Disease in pediatric patients recovering with high Vδ2+ T cells after allogeneic stem cell transplantation with unmanipulated bone marrow grafts: a prospective single-center cohort study

**DOI:** 10.3389/fimmu.2024.1433785

**Published:** 2024-07-29

**Authors:** Thilo Müller, Lina Alasfar, Friederike Preuß, Lisa Zimmermann, Mathias Streitz, Patrick Hundsdörfer, Angelika Eggert, Johannes Schulte, Arend von Stackelberg, Lena Oevermann

**Affiliations:** ^1^ Department of Pediatric Oncology and Hematology, Charité – Universitätsmedizin Berlin, Berlin, Germany; ^2^ Department of Internal Medicine V: Hematology, Oncology and Rheumatology, University Hospital Heidelberg, Heidelberg, Germany; ^3^ Department of Cardiology, Angiology and Intensive Care Medicine, German Heart Center Berlin, Berlin, Germany; ^4^ Department of Experimental Animal Facilities and Biorisk Management (ATB), Friedrich-Löffler-Institut, Greifswald, Germany; ^5^ Department of Pediatrics, Helios Klinikum Berlin-Buch, Berlin, Germany; ^6^ Department of Pediatrics I – Haematology, Oncology, Gastroenterology, Nephrology and Rheumatology, University Hospital Tübingen, Tübingen, Germany; ^7^ Berlin Institute of Health (BIH), Berlin, Germany

**Keywords:** pediatric stem cell transplantation, allogeneic stem cell transplantation, gamma delta (γδ) T cells, immune reconstitution, transplant immunobiology, Graft-versus-Host Disease (GVHD)

## Abstract

Gamma delta (γδ) T cells represent a minor fraction of human T cell repertoire but play an important role in mediating anti-infectious and anti-tumorous effects in the context of allogeneic hematopoietic stem cell transplantation (allo-HSCT). We performed a prospective study to analyze the effect of different transplant modalities on immune reconstitution of γδ T cells and subsets. CD3, CD4 and CD8 T cells were analyzed in parallel. Secondly, we examined the impact of γδ T cell reconstitution on clinical outcomes including acute Graft-versus-Host-Disease (aGvHD) and viral infections. Our cohort includes 49 pediatric patients who received unmanipulated bone marrow grafts from matched unrelated (MUD) or matched related (MRD) donors. The cohort includes patients with malignant as well as non-malignant diseases. Cell counts were measured using flow cytometry at 15, 30, 60, 100, 180 and 240 days after transplantation. Cells were stained for CD3, CD4, CD8, CD45, TCR*αβ*, TCRγδ, TCRVδ1, TCRVδ2, HLA-DR and combinations. Patients with a MRD showed significantly higher Vδ2+ T cells than those with MUD at timepoints +30, +60, +100 (p<0.001, respectively) and +180 (p<0.01) in univariate analysis. These results remained significant in multivariate analysis. Patients recovering with a high relative abundance of total γδ T cells and Vδ2+ T cells had a significantly lower cumulative incidence of grade II-IV aGvHD after transplantation (p=0.03 and p=0.04, respectively). A high relative abundance of Vδ2+ T cells was also associated with a lower incidence of EBV infection (p=0.02). Patients with EBV infection on the other hand showed higher absolute Vδ1+ T cell counts at days +100 and +180 after transplantation (p=0.046 and 0.038, respectively) than those without EBV infection. This result remained significant in a multivariate time-averaged analysis (q<0.1). Our results suggest a protective role of γδ T cells and especially Vδ2+ T cell subset against the development of aGvHD and EBV infection after pediatric HSCT. Vδ1+ T cells might be involved in the immune response after EBV infection. Our results encourage further research on γδ T cells as prognostic markers after HSCT and as possible targets of adoptive T cell transfer strategies.

## Introduction

Allogeneic hematopoietic stem cell transplantation (HSCT) is a curative treatment for various malignant and nonmalignant diseases. Delayed immune reconstitution (IR) is a main risk factor for morbidity and mortality in patients undergoing allogeneic HSCT as it is associated with higher rates of relapse, infectious complications and Graft-versus-Host-Disease (GvHD) (1). To further improve transplant-related outcomes in the future, it is critical to identify the factors that influence speed and quality of immune recovery after HSCT.

In the past two decades, more focus has been placed on the reconstitution of gamma delta (γδ) T cells as growing evidence suggests that these cells have beneficial effects in the context of HSCT, by mediating innate and adaptive immune responses independent of HLA-antigen presentation and by exerting potent antitumor activity via various receptors e.g. NKG2D or DNAM-1 (2).

While the majority of circulating CD3 lymphocytes carries an αβ T cell receptor, only 1-10% in the peripheral blood are γδ T cells. The γδ T cell receptor consists of γ and δ chains that are encoded by 6 Vγ genes on chromosome 6 respectively 8 Vδ genes on chromosome 14 (3). γδ T cells are subclassified based on their Vδ chain; Vδ2+ T cells are the predominant fraction found in the peripheral blood of healthy adults, whereas non-Vδ2+ T cells (mainly Vδ1+) are primarily found in epithelial tissue like skin or intestines (4, 5).

Nowadays, peripheral blood stem cells represent the main stem cell source for HSCT in adults. In contrast, unmanipulated bone marrow grafts among peripheral blood stem cells and cord blood are still frequently used in children. In addition to graft source and graft manipulation, donor selection has an important impact on IR.

In a recent meta-analysis of 11 studies (919 patients) on γδ T cell reconstitution after allogeneic HSCT, Arruda et al. reported that high γδ T cell counts were associated with less disease relapse, fewer viral infections and higher overall and disease-free survival (6). Most of these studies included only adult patients with partially mismatched related donors (PMRD). There is only few data on IR of γδ T cells and especially their subsets in children undergoing allogeneic HSCT from matched unrelated (MUD) or matched related donors (MRD).

In our prospective single-center cohort study, we report on the reconstitution of γδ T cells and subsets until day +240 after transplantation in a cohort of pediatric patients receiving unmanipulated bone marrow grafts. We studied the impact of transplant modalities on the IR and the effect of high versus low γδ T cells on HSCT outcomes.

## Material and methods

### Patients

This study includes 49 patients who underwent their first allogeneic HSCT between August 2016 and January 2019 at the Department of Pediatric Oncology and Hematology, *Charité – Universitätsmedizin Berlin*. The median patient age at HSCT was 7 years (0-19 years). All patients received unmanipulated bone marrow as graft source. The cohort consisted of 29 patients with malignant hematological disorders and 20 patients with various non-malignant HSCT-indications, mostly hemoglobinopathies. Detailed transplant characteristics of our cohort are presented in **Table 1**. Patients received different conditioning regimens dependent on the transplant indication (**Supplementary Figure 1**).

**Table 1:** Patient and transplant characteristics.

Number of patients		49
Follow-Up Time, days, median [IQR]		737 [474, 873]
Patient age, years, median [range]		7 [0 - 19]
Patient sex, n (percent)	Female	23 (46.9)
Disease, n (percent)	Acute lymphoblastic leukemia (ALL)	21 (42.9)
	Acute myeloid leukemia (AML)	6 (12.2)
	Myelodysplastic syndrome (MDS)	2 (4.1)
	Sickle cell disease (SCD)	10 (20.4)
	ß-Thalassemia	3 (6.1)
	Chronic granulomatous disease (CGD)	1 (2.0)
	Severe combined immunodeficiency (SCID)	1 (2.0)
	Severe aplastic anemia (SAA)	1 (2.0)
	Hb-Yokohama	1 (2.0)
	Fanconi anemia	1 (2.0)
	Congenital amegakaryocytic thrombocytopenia (CAMT)	1 (2.0)
	Osteopetrosis	1 (2.0)
Donor type, n (percent)	MSD	26 (53.1)
	MUD	20 (40.8)
	MFD	3 (6.1)
Antithymocyte globulin, n (percent)	Yes	33 (67.3)
Alemtuzumab, n (percent)	Yes	3 (6.1)
Methotrexat, n (percent)	Yes	36 (73.5)
Mycophenolatmofetil, n (percent)	Yes	14 (28.6)
Disease Status, n (percent)	Non-malignant disease	20 (40.8)
	Malignant disease	29 (59.2)
HLA Compability, n (percent)	9/10	3 (6.1)
	10/10	46 (93.9)
Conditioning Regimen, n (percent)	VP16/TBI	9 (18.4)
	Flu/TT/Treo	23 (46.9)
	Flu/TT/Mel	5 (10.2)
	Flu/Bu/TT	1 (2.0)
	Flu/TT	2 (4.1)
	Flu/Cy	2 (4.1)
	Flu/Bu/Cy/TT	2 (4.1)
	Flu/Bu	2 (4.1)
	Bu/Cy/Mel	2 (4.1)
	Amsacrine/Flu/Cy/Ara-C/TBI	1 (2.0)
Graft CD3, cells/kg, median [IQR]		4,9 x 10^7^ [2,8 x 10^7^, 7,3 x 10^7^]
Graft CD34, cells/kg, median [IQR]		5,2 x 10^6^ [3,4 x 10^6^, 7,8 x 10^6^]
Graft CD45, cells/kg, median [IQR]		3,9 x 10^8^ [2,9 x 10^8^, 5,8 x 10^8^]
CMV Serostatus, D/R, n (percent)	+/+	19 (44.2)
	+/-	7 (16.3)
	-/-	9 (20.9)
	-/+	8 (18.6)
Donor age, years, median [range]		14 [2 - 52]
Sex (mis-)match, n (percent)	F/F	12 (25.0)
	F/M	10 (20.8)
	M/F	9 (18.8)
	M/M	17 (35.4)

For GvHD-prophylaxis all patients received Ciclosporin A (CSA) intravenously twice daily starting from one day prior to transplantation. Patients were switched to oral CSA formulations before discharge from the hospital. In combination with CSA patients received either Mycophenolate Mofetil (MMF) 2x600 mg/m^2^/d starting on day +1 after HSCT for 30 days or Methotrexate (MTX) 10 mg/m^2^ once a day on days +1, +3 and +6.

Serotherapy was administered in 36 cases, 33 patients were treated with anti-thymocyte globulin (ATG) and 3 patients received Alemtuzumab (for detailed information on dosing and administration of serotherapy see **Supplementary Figure 1**).

In nine patients, immunosuppression with CSA was changed to Everolimus and MMF after transplantation. For one patient, this became necessary because he developed posterior reversible encephalopathy syndrome, while the other patients had acute renal failure.

### Ethics

Written informed consent was obtained from all patients or their parents/guardians before HSCT. The study was approved by the local ethics committee (EA2/144/15).

### Evaluation

The day of engraftment was defined as the first of three consecutive days with an absolute neutrophil count of at least 500 cells/µl.

In patients with leukemia, relapse was defined either morphologically as more than 5% blast cells in the bone marrow or a minimal residual disease of ≥ 1x10^-4^ measured by flow cytometry or polymerase chain reaction. Relapse of Myelodysplastic syndrome (MDS) was defined by morphology, cytogenetics, or both.

Acute GvHD was defined and diagnosed according to the modified Glucksberg criteria based on clinical, laboratory and histological findings (7, 8).

All patients were screened twice weekly for Cytomegalovirus (CMV), Epstein-Barr virus (EBV) and Adenovirus (ADV) DNA in peripheral blood and ADV DNA in stool with polymerase chain reaction until discharge. After discharge, analysis was performed once weekly. PCR cut-off levels for detection of EBV, CMV and ADV in blood were 550, 300 and 2000 Copies/ml, respectively. A linear range of the copy numbers was provided by the local laboratory between 1000 – 2,2x10^8^ Copies/ml (EBV), 2000 – 3x10^8^ (CMV) and 2000 – 1x10^8^ (ADV). The viral load in positive stool samples was measured semi-quantitatively.

### Sample collection

Whole blood samples (3 ml) from all patients undergoing HSCT were collected on ethylenediamine tetra acetic tubes and analyzed the same day at seven different timepoints: once prior to the start of the conditioning regimen and at six timepoints after HSCT on days +15, +30, +60, +100, +180, +240.

### Flow cytometry

Flow cytometry was performed using Duraclone technology (Beckman Coulter) and each timepoint was analyzed using the DuraClone IM Phenotyping T cell subtypes panel containing nine conjugated antibodies. Samples were processed according to validated standard operation procedures. All analyses were performed using a NAVIOS flow cytometer (Beckman Coulter). Measurements were performed according to the validated method described previously (9) and analyzed using the FlowJo 10.4.2 software. Cell counts were conducted for cells expressing CD3, CD4, CD8, CD45, TCRαβ, TCRγδ, TCRVδ1, TCRVδ2, HLA-DR and combinations.

### Statistical analysis

Wilcoxon rank test was performed for non-parametric variables, while comparing two outcomes or timepoints. For multiple strata or groups, the Kruskal test was performed. As a measure of effect size Cliffs Delta was computed for variables having two levels and Spearman correlation for continuous variables. When Spearman correlation was computed, the associated Spearman test of correlation was performed (10). Testing for confounded variables was done using a linear model with multiple variables and interaction terms followed by a log-likelihood ratio test with the null hypothesis being a single variable model. A significant log-likelihood ratio test indicates that the variable in question is confounded.

The analysis of outcomes in relation to the qualitative cell counts of interest i.e. high versus low was performed using the Cox hazard regression model via the R survival package (11) in accordance with the EBMT recommendations (12). To test for the statistical significance of the Cox fits, Rank-sum tests were performed.

The stratification of the cohort into patients with high and low cell relative abundance was done by ordering the patients by their time-averaged relative abundance of the cell type of interest, then finding the best cut-off value of this relative abundance that separates the cohort into two groups. This is defined by the largest effect size ω^2 comparing the Jaccard distances in the space of the time-averaged relative abundance and the rank of the patient based on it, **Supplementary Figure 2**.

In all tests, a p-value of 0.05 is considered significant for single-variable analysis. For multivariate analysis, the p-values were corrected for multiple testing using the Benjamini-Hochberg method (13) to get q-values. A corrected q-value of <0.1 is considered significant.

The t-distributed stochastic neighbor embedding (t-SNE) analysis was performed on a maximum of 200K cells randomly sampled from the samples corresponding to each timepoint and each group (grade II-IV GvHD or not). The analysis is performed using FlowJo software, with the coloring of cells corresponding to the gating performed.

Statistical analysis were performed using RStudio, Version 2023.06.2 + 561 (RStudio: Integrated Development Environment for R. Posit Software, PBC, Boston, MA).

## Results

### Patient outcomes

The median (Interquartile range) follow-up time in our cohort was 737 (474 – 873) days. Successful engraftment was achieved in 100% of the patients and none of them experienced graft rejection afterwards. During the follow-up period five patients (10%) died; three of the deaths were treatment-related mortalities (TRM); two patients died from relapse. Patients in the TRM group had severe infectious complications with consecutive multiple organ failure.

Relapse occurred in 13 of 29 cases (44.8%) in the subgroup of patients with malignant disease at a median of 171 days after transplantation. We registered 23 cases of aGvHD (46%). In eight of these patients (16%) higher grade aGvHD (grade II-IV) was diagnosed. The median onset of aGvHD was 17 (15 – 31) days after transplantation. Viral infections occurred in 34 cases (69%) and 19 patients (55% of patients with viral infection) experienced infection with more than one virus (CMV, EBV or ADV) during the course of transplantation. A total of 14 patients developed CMV infection (28.5%) at a median of 28 (9 – 40) days after transplantation. EBV infection was diagnosed in 25 patients at a median of 50 (42 – 108) days after transplant. In 13 patients ADV infection was diagnosed (26%) by positive ADV PCR from a stool sample. In eight of these patients, we also detected ADV replication in the blood. The median onset of systemic adenovirus infection was 24 (16 – 43) days after transplantation.

### Descriptive analysis of γδ T cell immune reconstitution after HSCT

CD3 cells quadrupled between day +30 and day +240 after transplantation. CD8 cell counts exceeded CD4 counts during the first 240 days after transplantation with the lowest CD4/CD8 ratio of 0.3 at day +100 after transplantation. Absolute and relative cell counts of T cells and their subsets are summarized in **Table 2**.

**Table 2:** Absolute and relative concentrations of T cells and subsets during the first 240 days after transplantation.

	Day +15	Day +30	Day +60	Day +100	Day +180	Day +240
Cell subset concentration (cells/µl), median (IQR)						
CD3	6 [0, 43]	297 [112, 593]	620 [251, 1026]	486 [335, 1121]	1011 [668, 1693]	1202 [879, 1754]
CD4	2.09 [0.20, 25.45]	112 [31, 241]	157 [75, 239]	153 [108, 224]	365 [236, 511]	469 [336, 612]
CD8	2.51 [0.08, 17.06]	156 [38, 288]	451 [102, 792]	316 [111, 827]	407 [286, 1089]	539 [326, 987]
αβ T cells	4 [0, 37]	280 [106, 565]	610 [239, 915]	459 [300, 1041]	943 [609, 1526]	1152 [791, 1551]
γδ T cells	1 [0, 5]	10 [2, 29]	17 [6, 49]	27 [14, 71]	58 [41, 94]	68 [42, 141]
Vδ1	0 [0, 1]	2 [0, 6]	4 [2, 15]	13 [3, 36]	29 [12, 53]	34 [17, 53]
Vδ2	0 [0, 3]	4 [1, 20]	4 [1, 22]	5 [2, 18]	14 [4, 23]	19 [8, 33]
nonVδ1-nonVδ2	0 [0, 0]	1 [0, 3]	3 [1, 7]	4 [1, 14]	9 [5, 17]	12 [6, 21]
Relative concentrations, %						
CD4/CD3	49 [19, 56.5]	36 [20.5, 48.2]	28 [14, 52.5]	29 [16, 48]	38 [25, 60]	41 [25, 55]
CD8/CD3	32 [19.3, 43.8]	55 [34.3, 70.3]	68.5 [38.8, 78.8]	66 [43, 73]	55 [32, 67]	52 [38.3, 64.5]
αβ T cells/CD3	84.5 [64, 94]	96 [92, 97.3]	95 [91.3, 98]	95 [90, 97.5]	94 [91, 96]	93.5 [91, 95]
γδ T cells/CD3	14.5 [4.3, 27]	4 [2.8, 8]	4.5 [2, 8]	5 [2, 10]	6 [4, 8]	6.5 [5, 9]
Vδ1/γδ T cells	20 [3.5, 35.8]	26.5 [11, 41.8]	41 [19, 48.8]	50 [27, 61.5]	48 [29, 67]	51 [35, 65.8]
Vδ2/γδ T cells	65 [39.5, 81]	60.5 [34.8, 83]	38 [16.3, 61.3]	25 [10, 51.5]	26 [8, 50]	26.5 [9, 48.5]
nonVδ1-nonVδ2/γδ T cells	5 [0, 12.5]	7 [2.6, 14.3]	13.5 [7.3, 20.8]	16 [10, 23]	14 [10, 21]	14 [9, 19]

**Figure 1:**
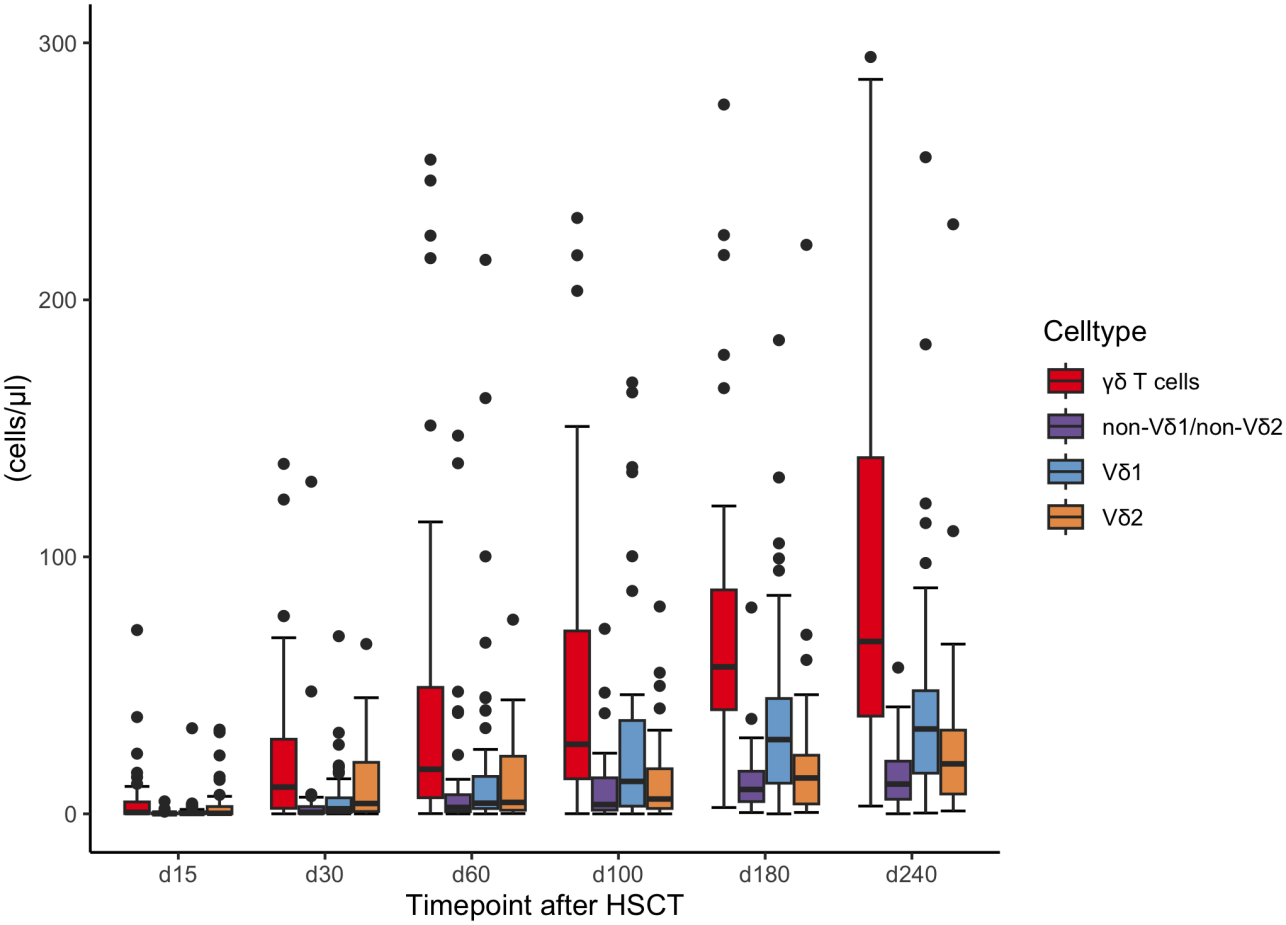
Concentrations of γδ T cells and subsets (Vδ1, Vδ2, non Vδ1-non Vδ2) during the first 240 days after transplantation. HSCT, Hematopoietic stem cell transplantation.

Counts of γδ T cells increased gradually after HSCT until day +240 after transplantation. Between day +30 and day +240 the proportion of γδ T cells of total CD3 cells was relatively stable between 4 and 6%. At day +240 the median count of γδ T cells (68/µl) was still lower than published reference values for healthy children (14). The Vδ2/Vδ1-ratio decreased continuously from day +30 to day +240 after transplantation and was < 1.0 after day +100, **Figure 1**.

### Impact of donor source on immune reconstitution

**Figure 2:**
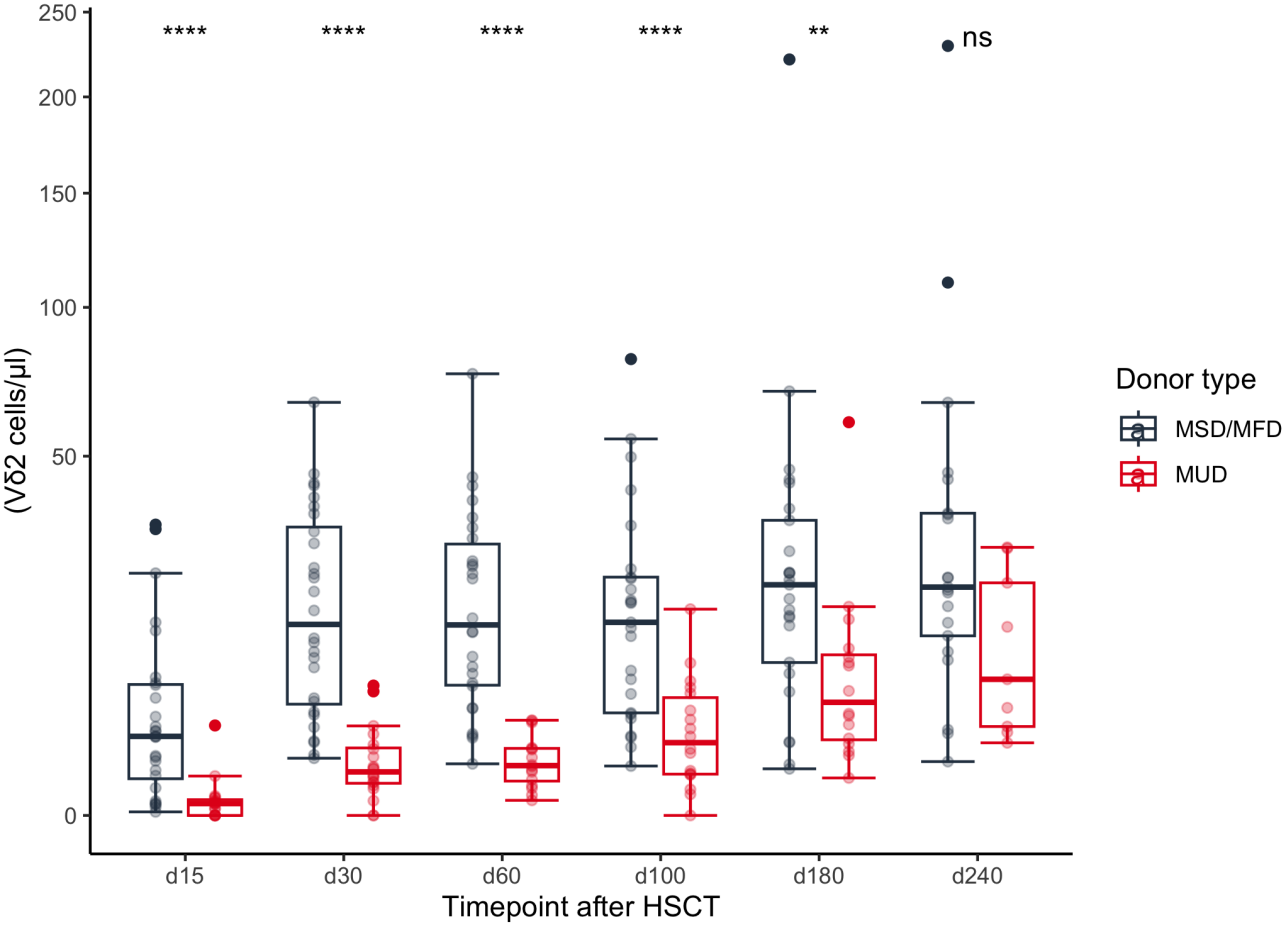
Concentrations of Vδ2 cells dependent on donor type during the first 240 days after transplantation. MSD, matched sibling donor; MFD, Matched family donor; MUD, Matched unrelated donor; HSCT, Hematopoietic stem cell transplantation. ** | p <= 0.01; **** | p <= 0.0001.

**Figure 3:**
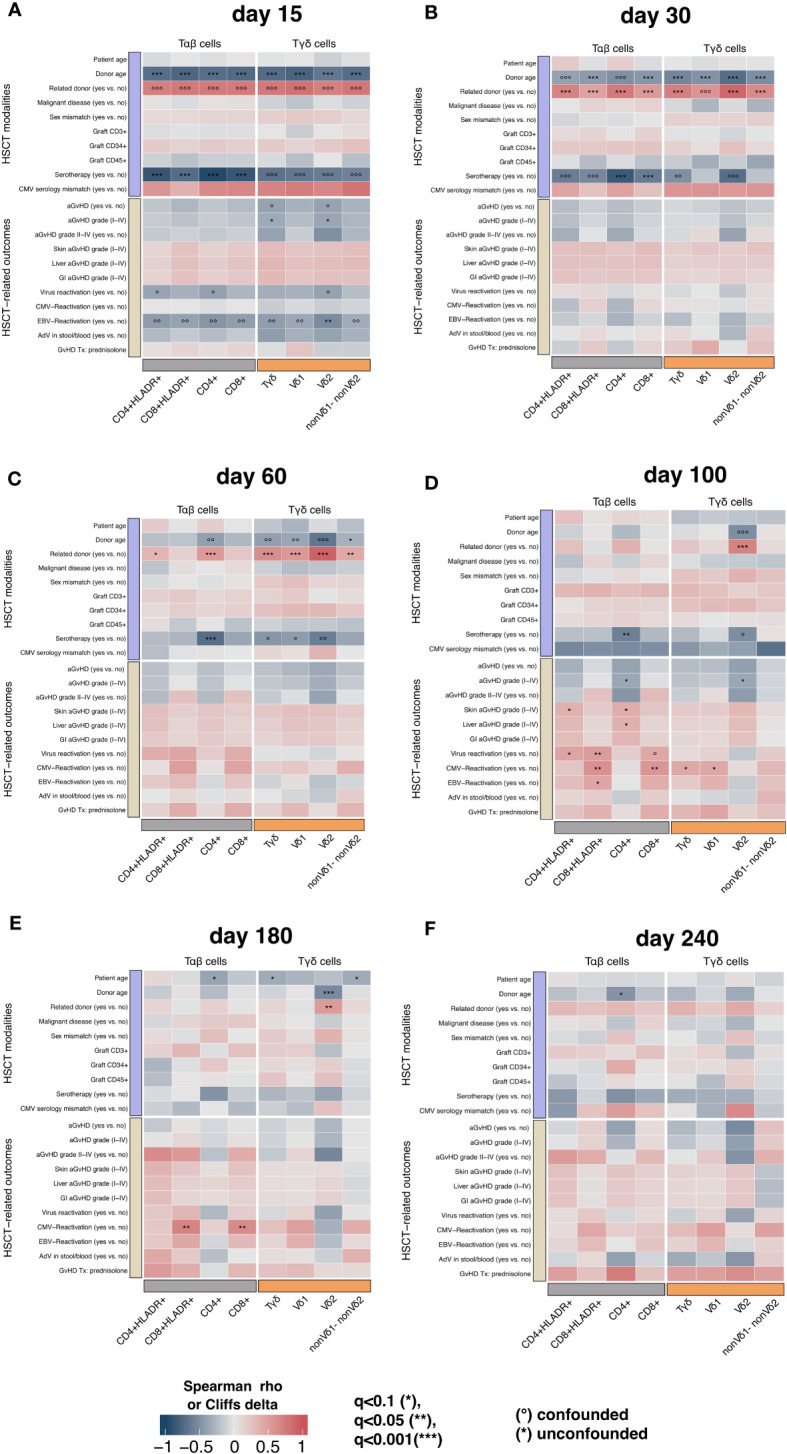
The correlation (in terms of spearman ρ or Cliffs δ) between T cell populations, transplant modalities and transplant outcomes at timepoints **(A)** day +15, **(B)** day +30, **(C)** day +60, **(D)** day +100, **(E)** day +180, **(F)** +240 with confounder analysis; confounded correlations are indicated with circles, while significant unconfounded are indicated with asterisk. A matched related donor (MSD or MFD) is the strongest transplantation modality correlating positively with γδ T cells and its subpopulations. HSCT, Hematopoietic stem cell transplantation; aGvHD, acute Graft-versus-Host-Disease; GI, gastro-intestinal.

**Figure 4:**
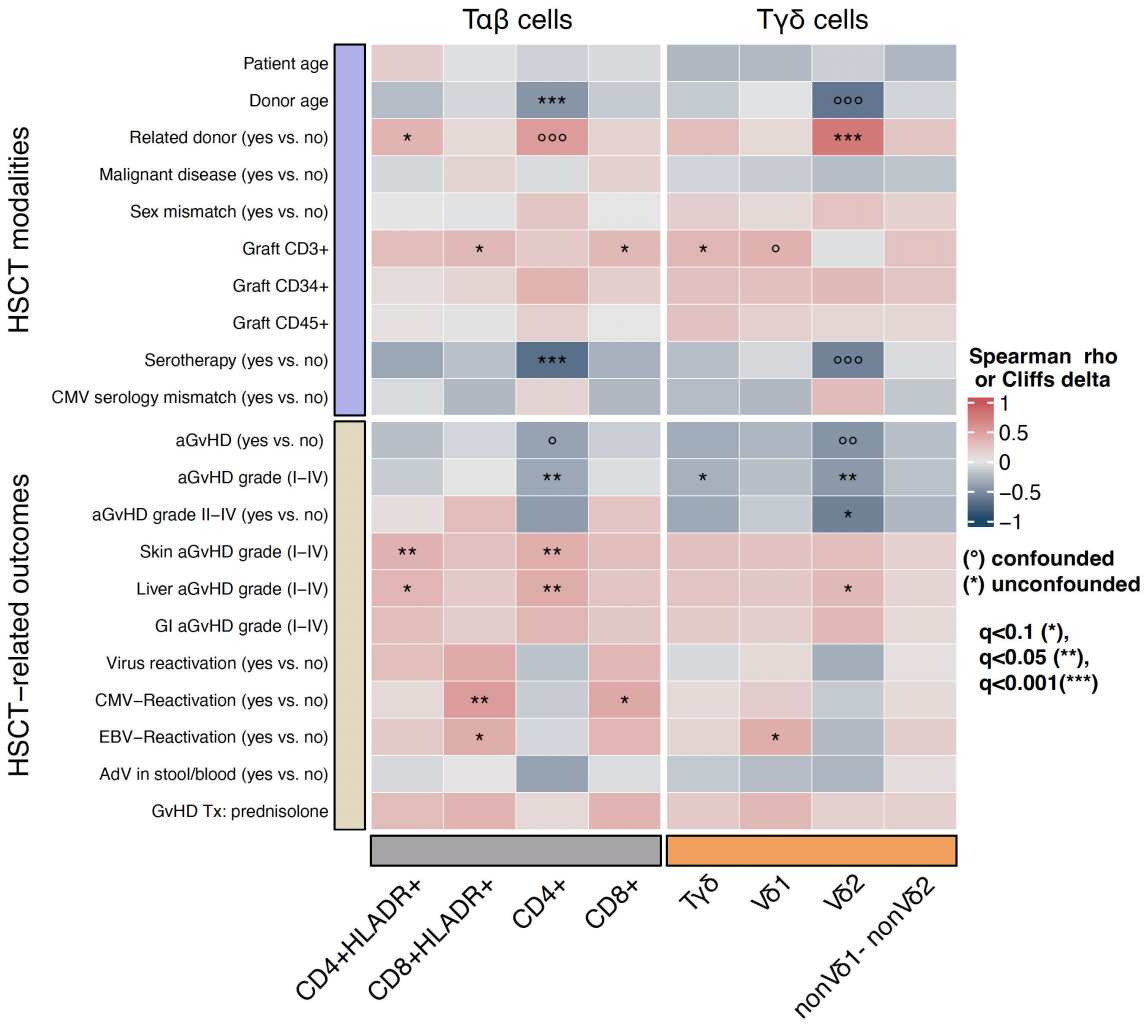
The correlation (in terms of spearman ρ or Cliffs δ) between the T cell populations, transplant modalities and transplant outcomes in a time-averaged approach, with confounder analysis, confounded correlations are indicated with circles, while significant unconfounded are indicated with asterisks. A matched related donor (MSD or MFD) is the strongest transplantation modality correlating positively with γδ T cells and its subpopulations. HSCT, Hematopoietic stem cell transplantation; aGvHD, acute Graft-versus-Host-Disease; GI, gastro-intestinal.

We analyzed the impact of pre-transplant modalities on γδ T cell reconstitution. In univariate analysis patients with a related donor (MSD or MFD) showed significantly higher absolute Vδ2+ T cell counts compared to patients with a matched unrelated donor (MUD) at timepoints +30, +60, +100 (p<0.0001, respectively) and +180 (p=0.004), **Figure 2**. In multivariate analyses this result remained significant for timepoints days +30, +60, +100 (q<0.001, respectively) and +180 (q<0.05), **Figure 3**. The correlation was also highly significant (q<0.001) in a multivariate time-averaged analysis, **Figure 4**.

The amount of total γδ T cells in patients with a related donor was higher at days +30 and +60 (q<0.001, respectively) after transplantation but not in the time-averaged analysis. The only timepoint with an association between elevated Vδ1+ T cells and MSD/MFD as donor source was day +60 (q<0.001) after transplantation, **Figure 3**. In summary, the donor type seems to especially influence Vδ2+ subtype reconstitution.

The donor type was also correlated with a faster recovery of the αβ T cell compartment in (uni-) and multivariate analysis. Early after transplantation (day +30) CD4 cells, HLA-DR positive CD4 cells, CD8 cells and HLA-DR positive CD8 cells showed faster recovery in patients with MSD or MFD (q<0.001, respectively). At day +60 after HSCT having a related donor was independently associated with higher CD4 (q<0.001) and CD4 HLA-DR positive cell counts (q<0.1), **Figure 3**. In the multivariate time-averaged analysis only CD4 HLA-DR positive cells showed a positive correlation with MSD/MFD donor status, **Figure 4**.

### Impact of other transplant modalities on immune reconstitution

In a multivariate analysis serotherapy with ATG or Alemtuzumab was independently associated with lower CD4 T cells at days +30, +60 (q<0.001, respectively) and +100 (q<0.05). This association was also highly significant in the multivariate time-averaged analysis (q<0,001). The same accounted for CD8 counts at day +30 (q<0.001). We saw no correlation between the use of serotherapy and the counts of total TCR γδ cells or subtypes. **Supplementary Figure 3** shows a subgroup analysis of Vδ2+ T cell reconstitution in patients with malignant disease that received BM from a MRD without previous serotherapy compared to patients with sickle cell disease that received ATG before transplant from a MRD. Both groups presented high Vδ2+ T cell counts early after transplantation in contrast to patients with a MUD, highlighting serotherapy did not affect γδ T cell reconstitution in our cohort.

Higher donor age (age as a continuous variable) was associated with lower counts of total γδ, Vδ1+, Vδ2+, non-Vδ1/non-Vδ2 T cells (q<0,001, respectively), CD8 HLA-DR+ and CD8 cells (q<0,001, respectively) at day +30 and Vδ2+ T cells at day +180 (p<0.001). Donor age was also negatively associated with CD4 counts at day +240 (q<0.1) and in the time-averaged analysis (q<0,001), **Figures 3**, **4**.

### Immune reconstitution and clinical outcomes

#### Death and relapse

There was no difference in cumulative incidence of death between patients with high versus low TCR γδ cells and Vδ2+ or Vδ1+ subsets respectively. In the subgroup of patients with malignant hematological disorders we observed no statistical difference of relapse incidence between the two groups.

#### Acute Graft-versus-Host-Disease

**Figure 5:**
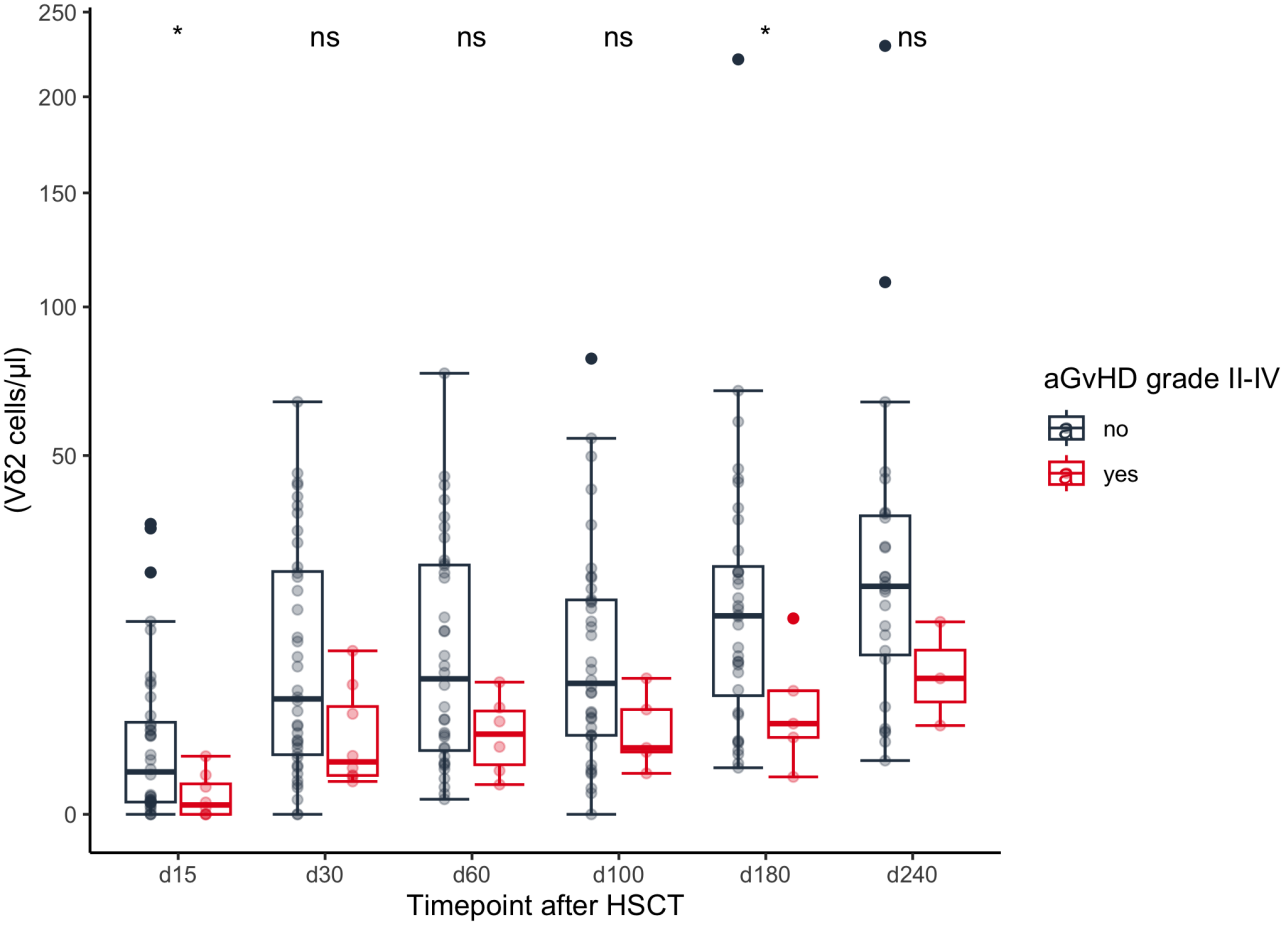
Comparison of absolute Vδ2+ T cell counts between patients with and without grade II-IV aGvHD. HSCT, Hematopoietic stem cell transplantation; aGvHD, acute Graft-versus-Host-Disease. ns | not significant; * | p <= 0.05.

In univariate analysis patients without aGvHD (grade I-IV) showed higher absolute median Vδ2+ T cell counts at day +60 (p=0.048), day +100 (p=0.023) and day +240 (p=0.027) compared to those with aGvHD (data not shown). Patients with higher grade aGvHD (grade II-IV) had higher Vδ2+ T cell counts at day +180 (p = 0.046), **Figure 5**. This result remained significant in multivariate time-averaged analysis (q<0.1), but not at single timepoints, **Figures 3**, **4**. The grade of aGvHD was independently negatively correlated with the Vδ2+ T cell counts at day +100 (q<0.1) and in multivariate time-averaged analysis (q<0.05), **Figures 3**, **4**. We observed no difference for total γδ T cells and Vδ1+ subset.

**Figure 6:**
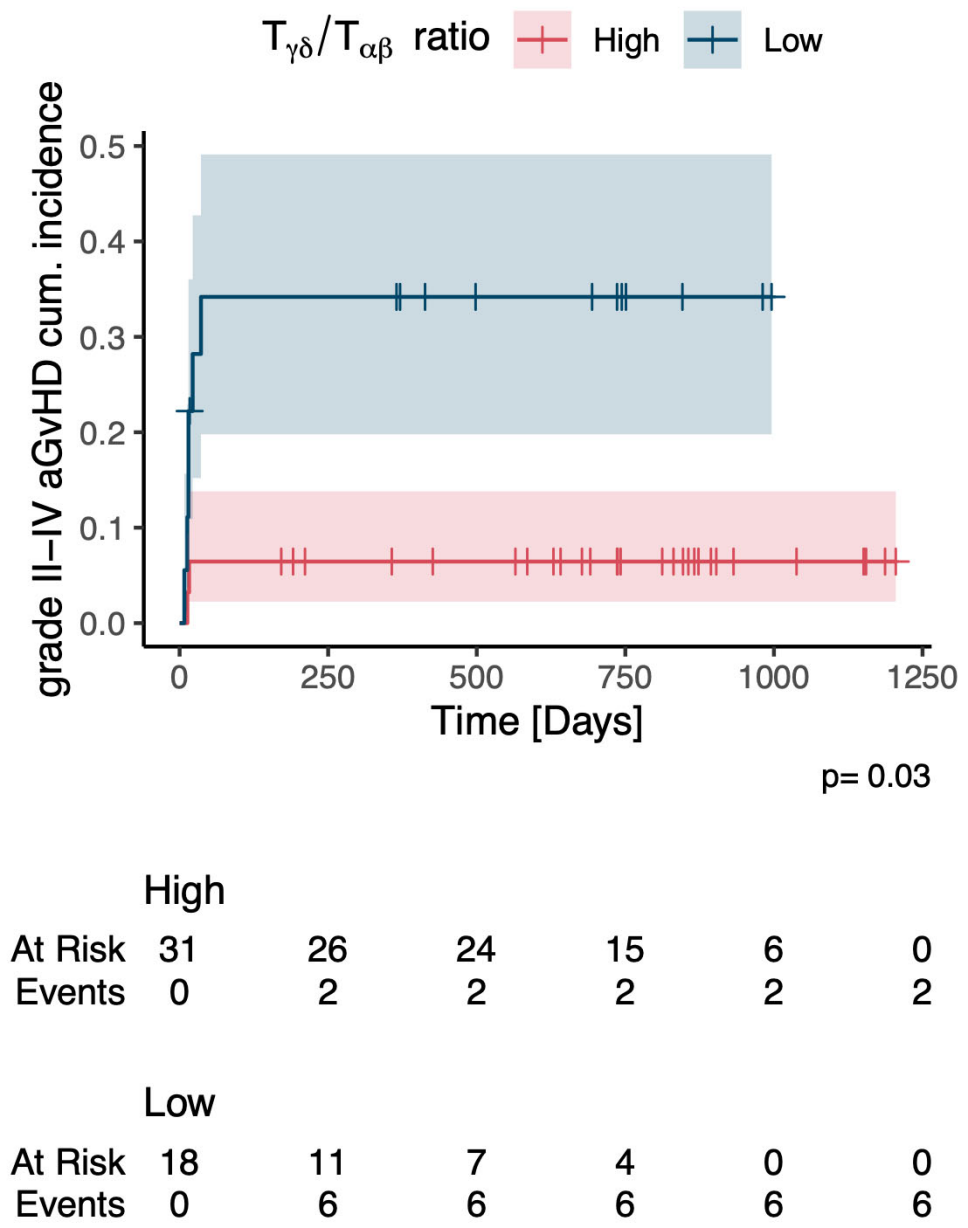
Cumulative incidence of grade II-IV acute GvHD in patients with a high vs. low relative abundance of γδ T cells. aGvHD, acute Graft-versus-Host-Disease.

**Figure 7:**
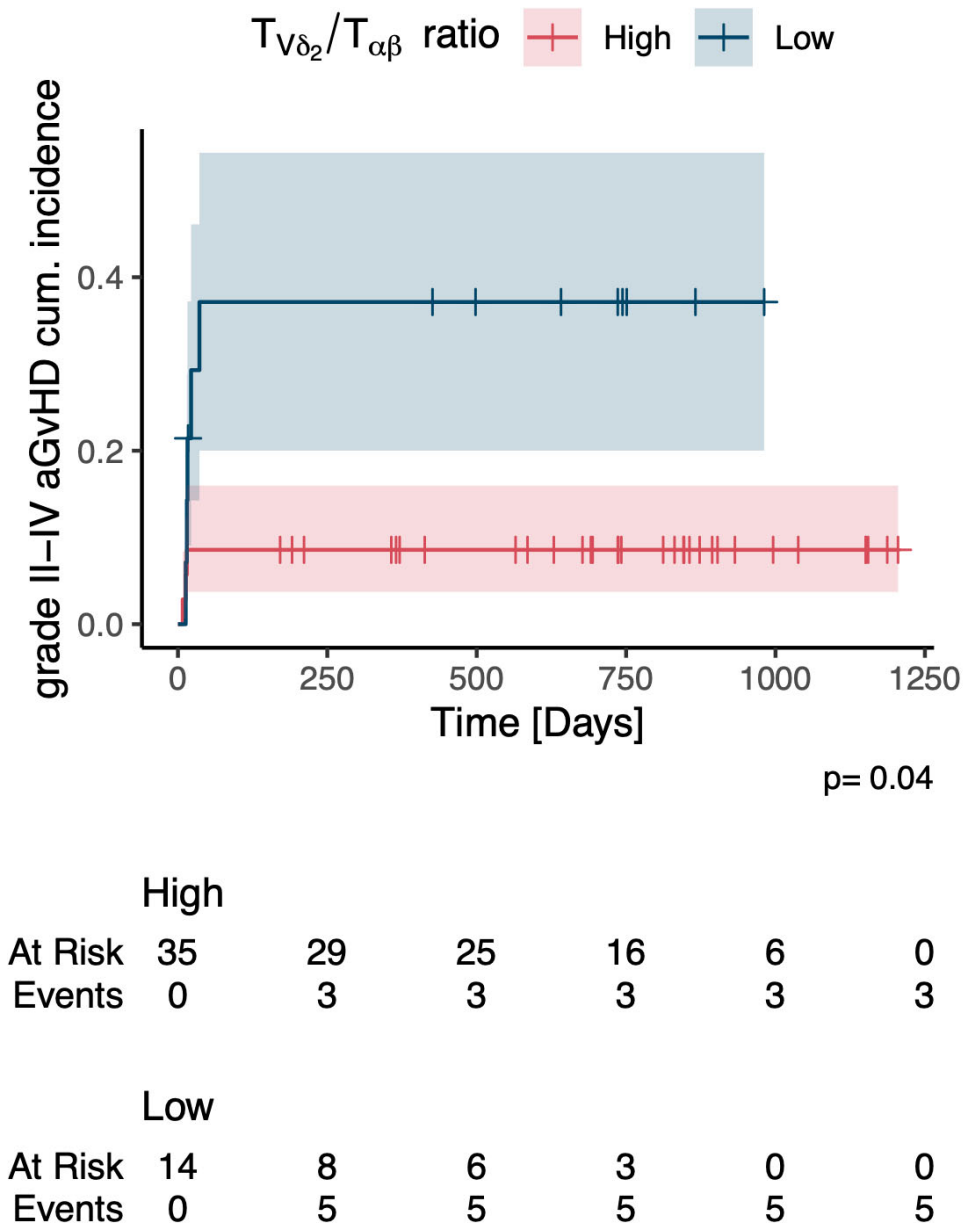
Cumulative incidence of grade II-IV acute GvHD in patients with a high vs. low relative abundance of Vδ2+ T cells. aGvHD, acute Graft-versus-Host-Disease.

**Figure 8:**
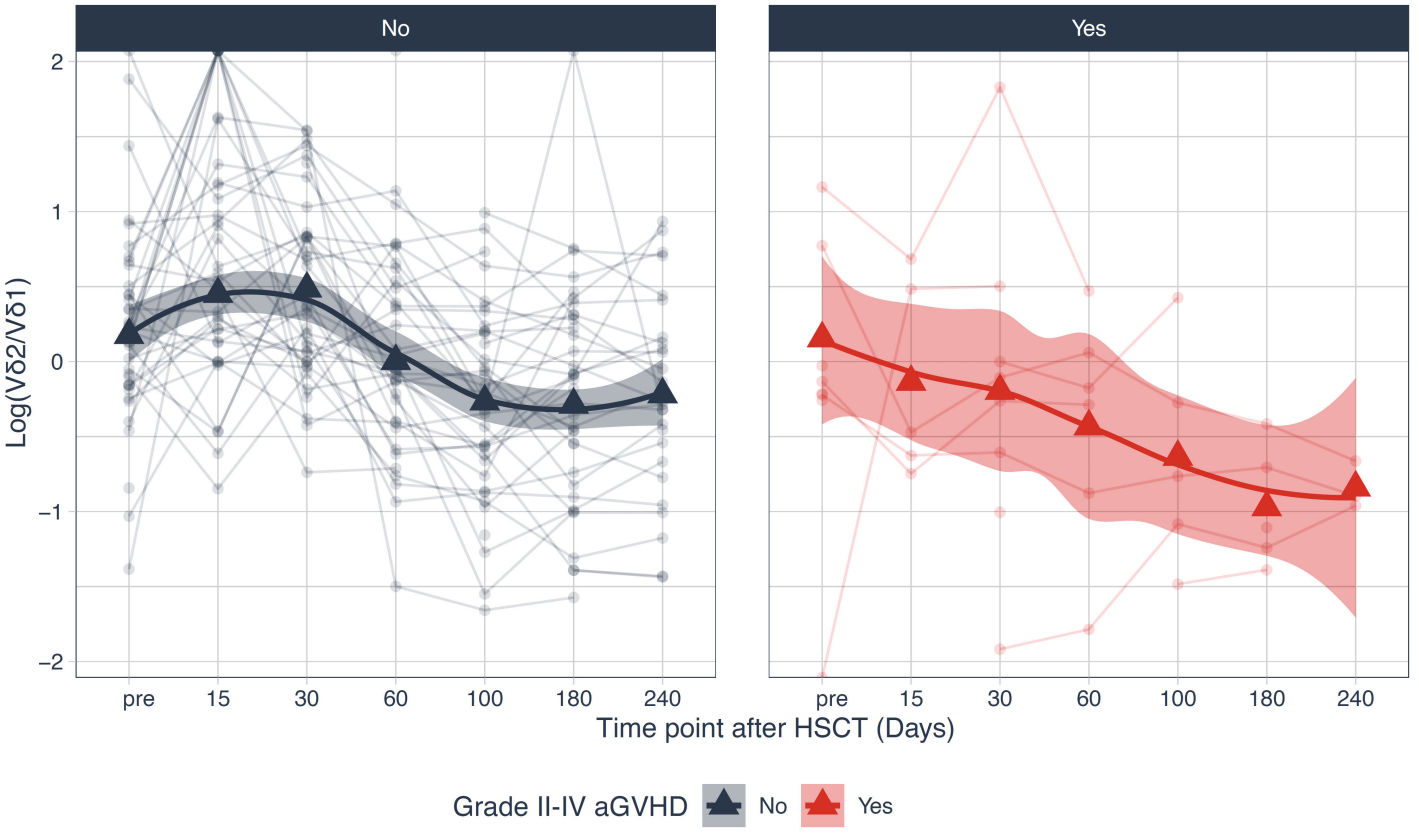
Logarithm of the Vδ2/Vδ1-ratio stratified over patients with and without grade II-IV aGvHD. HSCT, Hematopoietic stem cell transplantation; aGvHD, acute Graft-versus-Host-Disease.

In a multivariate time-averaged analysis the grade of aGvHD was independently negatively correlated with the Vδ2+ T cell count (q<0.05), **Figure 4**. Patients with a high relative abundance of total γδ T cells (p=0.03) and Vδ2+ T cells (p=0.04) had a significantly lower cumulative incidence of grade II-IV aGvHD, **Figures 6**, **7**. Patients with and without grade II-IV aGvHD showed a population inversion concerning the Vδ2/Vδ1-ratio after HSCT. While patients without grade II-IV aGvHD showed a trend towards recovering pre-HSCT Vδ2/Vδ1-ratio, those with grade II-IV aGvHD had an ongoing decline until day +240 after HSCT, **Figure 8**.

We observed a lower cumulative incidence of aGvHD of the skin in patients with high total TCR γδ cells compared to those with low numbers (p=0.02), **Supplementary Figure 4**. No association was found for aGvHD of the liver or the gut.

**Figure 9:**
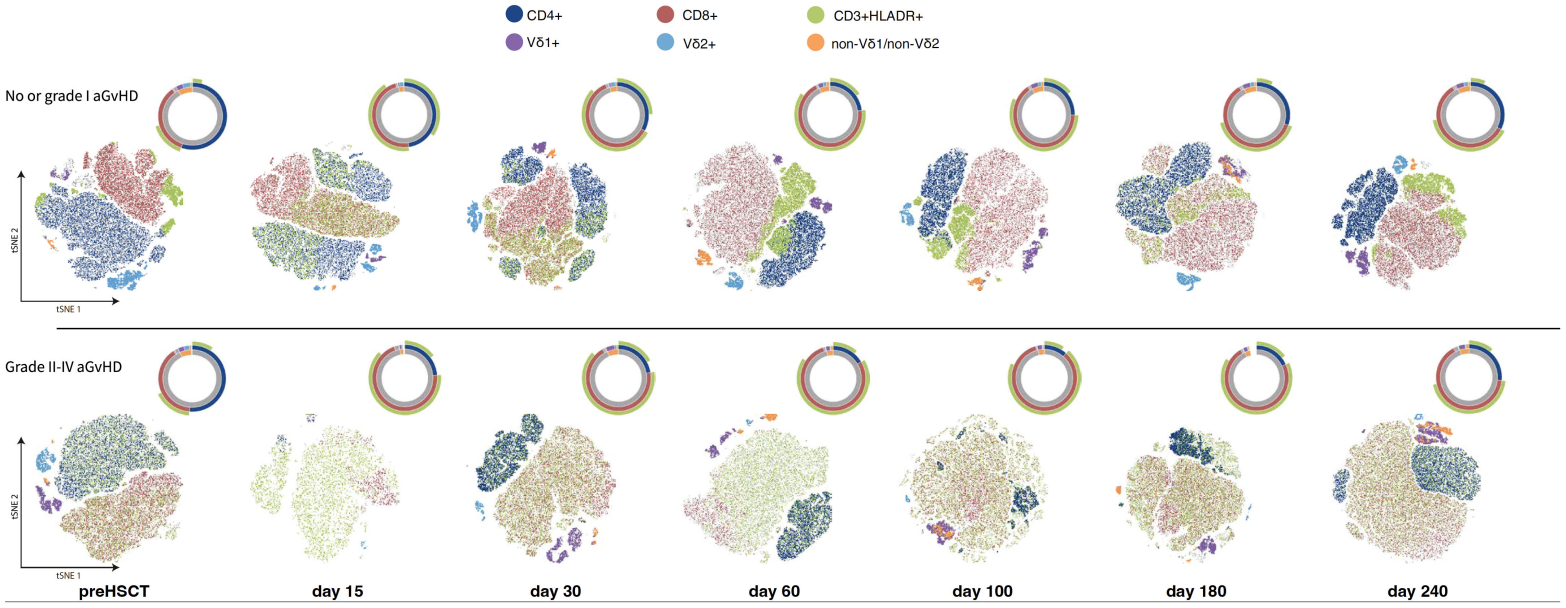
t-distributed stochastic neighbor embedding (t-SNE) dimensionality reduction and clustering of the CD3 cells, while the color indicates the gating performed on those cells. The cell populations are randomly subsampled from all the stratified samples according to the aGvHD grade status (no or grade I versus grade II-IV aGvHD) to a maximum of 200K events per time point. The counts of the gated T cell subpopulations are shown qualitatively in the sunburst diagrams.

**Figure 9** illustrates the impact of aGvHD on immune reconstitution by using t-distributed stochastic neighbor embedding (t-SNE) dimensionality reduction and clustering of CD3 lymphocytes. Patients are stratified according to the grade of aGvHD (no or grade I aGvHD vs. grade II-IV aGvHD). The CD4/CD8 ratio is smaller in patients with grade II-IV aGvHD and they show a higher proportion of activated HLA-DR positive lymphocytes on days +180 and +240. The fraction of Vδ2+ T cells is diminished in patients with higher grade aGvHD while the Vδ1+ and non-Vδ1/non-Vδ2 subset is present in both strata.

#### EBV infection

We examined the association between virus infection and γδ T cell reconstitution. In a first step we compared transplant modalities, HSCT outcomes and IR data of CD3, CD4, CD8, TCR αβ, TCR γδ, Vδ1, Vδ2 and non-Vδ1/non-Vδ2 T cells between patients with and without viral infections.

Patients with EBV infection had a higher median donor age (27.5 vs. 11 years, p=0.024) and more frequently received ATG (88% vs. 45.8%, p=0.004) or had an EBV-positive donor (100% vs. 68.2%, p=0.011) than patients without EBV infection. There was no significant difference concerning the distribution of underlying disease or conditioning regimens between the two groups. Rates of aGvHD and grade II-IV aGvHD were comparable between EBV-positive and EBV-negative patients (p=0.893 and 0.739, respectively). No significant difference was seen in CMV and ADV infection rates (p>0.2, respectively), **Table 3**.

**Table 3:** Distribution of transplant modalities and transplant outcomes between patients with and without EBV infection after transplantation.

		No EBV infection	EBV infection	p
n		24	25	
Diagnosis (%)	Acute lymphoblastic leukemia (ALL)	13 (54.2)	8 (32.0)	0.242
	Acute myeloid leukemia (AML)	2 (8.3)	4 (16.0)	
	Myelodysplastic syndrome (MDS)	2 (8.3)	0 (0.0)	
	Sickle cell disease (SCD)	5 (20.8)	5 (20.0)	
	ß-Thalassemia	0 (0.0)	3 (12.0)	
	Chronic granulomatous disease (CGD)	1 (4.2)	0 (0.0)	
	Severe combined immunodeficiency (SCID)	1 (4.2)	0 (0.0)	
	Severe aplastic anemia (SAA)	0 (0.0)	1 (4.0)	
	Hb-Yokohama	0 (0.0)	1 (4.0)	
	Fanconi anemia	0 (0.0)	1 (4.0)	
	Congenital amegakaryocytic thrombocytopenia (CAMT)	0 (0.0)	1 (4.0)	
	Osteopetrosis	0 (0.0)	1 (4.0)	
Donor type (%)	MSD	17 (70.8)	9 (36.0)	**0.027**
	MUD	7 (29.2)	13 (52.0)	
	MFD	0 (0.0)	3 (12.0)	
ATG (%)	No	13 (54.2)	3 (12.0)	**0.004**
	Yes	11 (45.8)	22 (88.0)	
Alemtuzumab (%)	No	22 (91.7)	24 (96.0)	0.971
	Yes	2 (8.3)	1 (4.0)	
Serotherapy (%)	No	11 (45.8)	2 (8.0)	**0.007**
	Yes	13 (54.2)	23 (92.0)	
Conditioning regimen (%)	VP16/TBI	3 (12.5)	6 (24.0)	0.198
	Flu/TT/Treo	14 (58.3)	9 (36.0)	
	Flu/TT/Mel	2 (8.3)	3 (12.0)	
	Flu/Bu/TT	0 (0.0)	1 (4.0)	
	Flu/TT	2 (8.3)	0 (0.0)	
	Flu/Cy	0 (0.0)	2 (8.0)	
	Flu/Bu/Cy/TT	0 (0.0)	2 (8.0)	
	Flu/Bu	2 (8.3)	0 (0.0)	
	Bu/Cy/Mel	1 (4.2)	1 (4.0)	
	Amsacrine/Flu/Cy/Ara-C/TBI	0 (0.0)	1 (4.0)	
Donor age (median [IQR])		11 [7, 24]	27 [11, 35]	**0.024**
EBV Status Donor (%)	negative	7 (31.8)	0 (0.0)	**0.011**
	positive	15 (68.2)	23 (100.0)	
EBV Status Patient (%)	negative	7 (31.8)	2 (9.1)	0.135
	positive	15 (68.2)	20 (90.9)	
aGVHD (%)	no	12 (50.0)	14 (56.0)	0.893
	yes	12 (50.0)	11 (44.0)	
aGvHD grade II-IV (%)	no	21 (87.5)	20 (80.0)	0.746
	yes	3 (12.5)	5 (20.0)	
CMV (%)	no	19 (79.2)	16 (64.0)	0.391
	yes	5 (20.8)	9 (36.0)	
ADV (%)	no	20 (83.3)	21 (84.0)	1.000
	yes	4 (16.7)	4 (16.0)	

Significant p-values are indicated in bold.

There was no difference for total γδ T cells and Vδ2+ T cells in the TCR γδ cell compartment at any timepoint, but Vδ1+ T cell counts were significantly higher at day +100 and day +180 in patients with EBV infection (p=0.046 and 0.038, respectively), **Supplementary Figure 5**. This association was even more distinct in the analysis of relative Vδ1+ T cell counts (in percent of total TCR γδ cells). At timepoints +60 (p=0.036), +100 (p=0.006), +180 (p=0.002) and +240 (p=0.009) EBV-positive patients had a significantly higher percentage of Vδ1+ T cells than EBV-negative patients, **Supplementary Figure 6**.

In multivariate analysis the association between elevated Vδ1+ T cell counts and EBV infection remained significant in the time-averaged model (q<0.1) but not at single timepoints, **Figures 3**, **4**.

**Figure 10:**
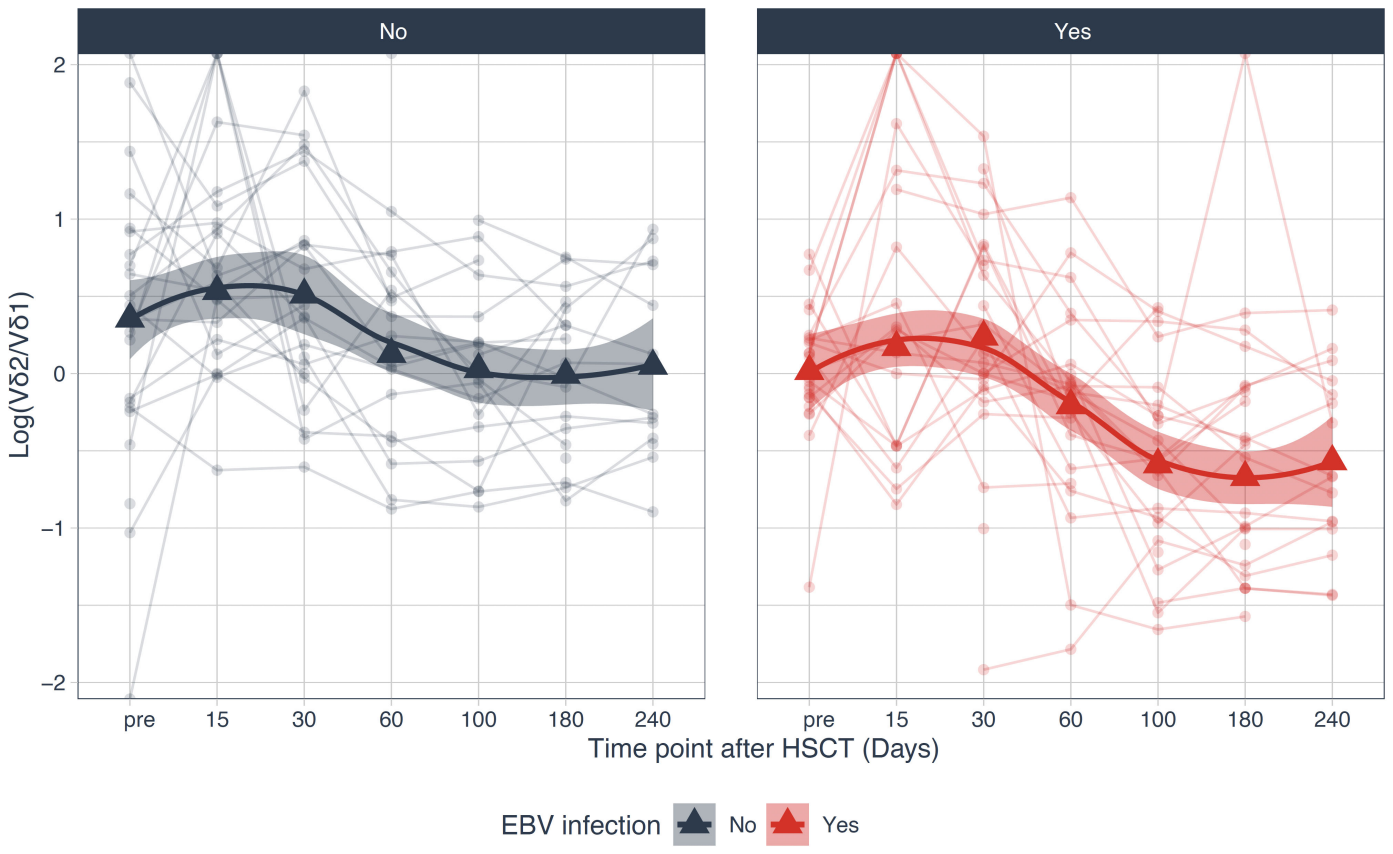
Logarithm of the Vδ2/Vδ1-ratio stratified over patients with and without EBV infection. HSCT, Hematopoietic stem cell transplantation.

As mentioned before, Vδ2+ T cell reconstitution was delayed in the whole cohort and especially in patients with grade II-IV aGvHD. We saw the same effect for patients with EBV infection. Vδ2/Vδ1-ratio decreased during the first 240 days after transplantation. In patients without EBV infection it stabilized and stayed >1, while in patients with EBV infection Vδ2/Vδ1-ratio continuously decreased from day +30 (1.5) until day +240 (0.2), **Figure 10**.

**Figure 11:**
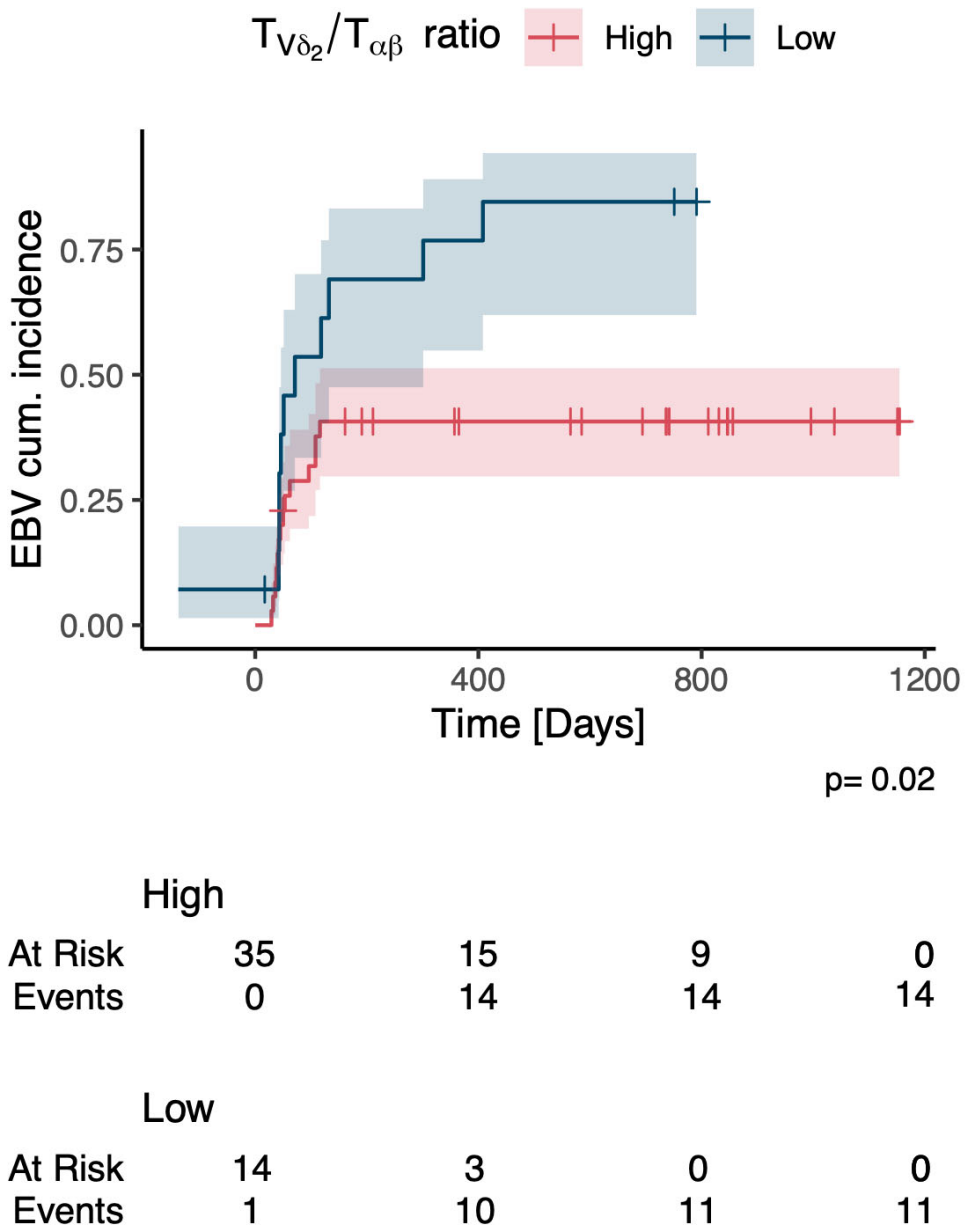
Cumulative incidence of EBV infection in patients with a high vs. low relative abundance of Vδ2 cells.

After stratification of the cohort into patients with high and low relative abundances of total TCR γδ, Vδ1+ and Vδ2+ T cells we registered a lower cumulative incidence of EBV-infection in patients with a high relative abundance of Vδ2+ T cells after HSCT compared to those with low Vδ2+ T cells (p=0.02), **Figure 11**.

#### CMV infection

Analogously, we studied the association of T cell subset reconstitution and the risk of CMV infection after HSCT. Patients with CMV infection had higher mortality (p=0.03) and higher rates of grade II-IV aGvHD compared to patients without CMV infection (p=0.006). The amount of CD45 positive cells in the bone marrow graft was significantly higher in the group without CMV infection (p=0.045). There was no difference concerning counts of CD3 and CD34 positive cells in the grafts and pre-transplant CMV status between the two groups, **Table 4**.

**Table 4:** Distribution of transplant modalities and transplant outcomes between patients with and without CMV infection after transplantation.

		No CMV infection	CMV infection	p
n		35	14	
Diagnosis (%)	Acute lymphoblastic leukemia (ALL)	14 (40.0)	7 (50.0)	0.174
	Acute myeloid leukemia (AML)	2 (5.7)	4 (28.6)	
	Myelodysplastic syndrome (MDS)	2 (5.7)	0 (0.0)	
	Sickle cell disease (SCD)	9 (25.7)	1 (7.1)	
	ß-Thalassemia	3 (8.6)	0 (0.0)	
	Chronic granulomatous disease (CGD)	1 (2.9)	0 (0.0)	
	Severe combined immunodeficiency (SCID)	1 (2.9)	0 (0.0)	
	Severe aplastic anemia (SAA)	1 (2.9)	0 (0.0)	
	Hb-Yokohama	1 (2.9)	0 (0.0)	
	Fanconi anemia	0 (0.0)	1 (7.1)	
	Congenital amegakaryocytic thrombocytopenia (CAMT)	0 (0.0)	1 (7.1)	
	Osteopetrosis	1 (2.9)	0 (0.0)	
Conditioning regimen (%)	VP16/TBI	5 (14.3)	4 (28.6)	0.183
	Flu/TT/Treo	18 (51.4)	5 (35.7)	
	Flu/TT/Mel	4 (11.4)	1 (7.1)	
	Flu/Bu/TT	1 (2.9)	0 (0.0)	
	Flu/TT	2 (5.7)	0 (0.0)	
	Flu/Cy	1 (2.9)	1 (7.1)	
	Flu/Bu/Cy/TT	2 (5.7)	0 (0.0)	
	Flu/Bu	2 (5.7)	0 (0.0)	
	Bu/Cy/Mel	0 (0.0)	2 (14.3)	
	Amsacrine/Flu/Cy/Ara-C/TBI	0 (0.0)	1 (7.1)	
Graft CD3, cells/kg (median [IQR])		5,6 x 10^7^ [3,2 x 10^7^, 7,4 x 10^7^]	4,3 x 10^7^ [2,5 x 10^7^, 5,6 x 10^7^]	0.168
Graft CD34, cells/kg (median [IQR])		5,6 x 10^6^ [3,5 x 10^6^, 8,0 x 10^6^]	4,2 x 10^6^ [3,2 x 10^6^, 5,9 x 10^6^]	0.212
Graft CD45, cells/kg (median [IQR])		4,4 x 10^8^ [3,1 x 10^8^, 6,2 x 10^8^]	3,1 x 10^8^ [2,0 x 10^8^, 4,5 x 10^8^]	**0.045**
CMV Status Donor (%)	negative	14 (42.4)	3 (21.4)	0.299
	positive	19 (57.6)	11 (78.6)	
CMV Status Patient (%)	negative	14 (43.8)	3 (23.1)	0.338
	positive	18 (56.2)	10 (76.9)	
Death (%)	no death	34 (97.1)	10 (71.4)	**0.030**
	death	1 (2.9)	4 (28.6)	
NRM (%)	no	35 (100.0)	11 (78.6)	**0.030**
	yes	0 (0.0)	3 (21.4)	
aGVHD (%)	no	20 (57.1)	6 (42.9)	0.556
	yes	15 (42.9)	8 (57.1)	
aGvHD grade II-IV (%)	no	33 (94.3)	8 (57.1)	**0.006**
	yes	2 (5.7)	6 (42.9)	
EBV (%)	no	19 (54.3)	5 (35.7)	0.391
	yes	16 (45.7)	9 (64.3)	
ADV (%)	no	31 (88.6)	10 (71.4)	0.299
	yes	4 (11.4)	4 (28.6)	

Significant p-values are indicated in bold.

Patients with CMV infection had significantly higher CD8 counts at days +60, +100 and + 180 after transplantation (p=0.026, p=0.006, p=0.006, respectively). The same accounted for CD3 positive cells at the same timepoints (p=0.03, p=0.012, p=0.005, respectively) while there was no difference at any timepoint for CD4 positive cells. In the γδ T cell compartment we observed significantly higher amounts of total γδ T cells at day +100 (p=0.038) and of Vδ1+ T cells at days +100 and +180 (p=0.038 and p=0.041, respectively), **Supplementary Figures 7-10**. No difference was seen for Vδ2+ T cells.

Relative counts of γδ T cell subsets significantly differed between patients with and without CMV infection at day + 180. Counts of Vδ1 cells (in percent of total TCR γδ cells) were higher in patients with CMV infection (p=0.008), while relative counts Vδ2+ T cells were decreased consecutively (p=0.004), data not shown.

In multivariate analysis including the impact of various pre-transplant modalities and transplant outcomes CD8 cells were still significantly higher in patients with CMV reactivation at days +100 and +180 (q<0,05, respectively). Total γδ T cells and Vδ1+ T cells were higher at day +100 (q<0,1, respectively). CD8 counts were also higher at day +180 (p<0.05). In the multivariate time-averaged model the result remained significant for CD8 cells, **Figures 3**, **4**.

After stratification into patients with high or low relative abundance of total TCR γδ, Vδ1+ and Vδ2+ T cells, we saw a lower cumulative incidence of CMV infection in patients with high total γδ T cells (p=0.02), **Supplementary Figure 11**. No difference was observed for patients with a high relative abundance of Vδ1+ or Vδ2+ T cells.

#### ADV infection

The group of patients that experienced systemic ADV infection (blood PCR positive) showed a significantly higher rate of aGvHD grade II-IV (p=0.022) and had been hospitalized significantly longer after HSCT than those patients without or with gastrointestinal ADV infection (66 days (60-88 days) vs. 48 days (44-58 days), p=0.009), data not shown.

We found no significant association between ADV infection (systemic as well as only gastrointestinal infection) and the reconstitution of any of the examined T cell subtypes.

## Discussion

In the past, various studies reported favorable outcomes in patients recovering with high γδ T cells after allogeneic HSCT, highlighting their potent anti-infectious and anti-tumorous abilities. Meanwhile data from pediatric cohorts and especially those with unmanipulated, HLA-matched grafts remain scarce. Despite promising results of transplant settings using haploidentical TCRab/CD19-depleted grafts in children (15), unmanipulated bone marrow grafts remain the standard of care for many transplant indications in the pediatric field. Both strategies differ fundamentally in terms of γδ T cell reconstitution due to the high content of these cells in the manipulated grafts. We performed a study on the dynamics and the clinical impact of γδ T cell reconstitution in the context of unmanipulated bone marrow transplantation.

One of the challenges in studying IR is defining thresholds for high and low γδ T cells. The definitions used in earlier studies differed widely and depended on the timepoint of assessment. Two studies defined high γδ T cell reconstitution as reaching a cut-off of 150 respectively 175 cells/µl at two consecutive timepoints in a timeframe of one year post HSCT (16, 17). Only about 10% of the included patients reached this cut-off, which bears the risk of an underestimated effect of robust γδ T cell reconstitution. A different approach is the use of median γδ T cell counts at certain timepoints for stratification. Minculescu et al. recently reported improved overall survival and relapse-free survival with lower incidence of aGvHD in adult patients with above median concentrations of γδ T cells at day +56 after HLA-matched, T cell replete stem cell transplantation (18). Other studies used relative cell counts at different single timepoints for stratification (19). Both strategies have their limitations as it is not clear which timepoint is most suitable for stratification because HCST complications occur at different stages after transplantation. Our approach to addressing the problem of stratification was using the time-averaged ratios R = TCR γδ/TCR αβ, Vδ2/TCR αβ and Vδ1/TCR αβ cells. The cut-off for high and low γδ T cells and subsets was found using clustering in the space of the ratio and the ranking of the patients based on it.

Few studies investigated the impact of graft sources on the reconstitution of γδ T cells. Perko et al. found significantly higher γδ T cell counts in patients with matched related donors (MRD) compared to matched unrelated donors (MUD) (17). Eyrich et al. described a subgroup of 12 pediatric patients that received bone marrow grafts from MSD in which some of the patients showed transient γδ T cell expansion early after transplantation, that did not occur in the other subgroup with CD34^+^ selected PBSC grafts from unrelated donors (20). Others reported faster γδ T cell reconstitution in MSD/MUD transplantation compared to cord blood (21) or in patients with αβ-depleted PBSC grafts compared to CD34^+^ selection (22).

In our study we investigated not only the correlation between donor source and reconstitution of total γδ T cells but also its subsets Vδ1+, Vδ2+ and non-Vδ1/non-Vδ2 T cells. We could demonstrate that especially Vδ2+ T cell reconstitution is severely hampered in patients with non-related donors while patients with MSD or MFD showed fast Vδ2+ T cell recovery early after transplantation. This finding was independent of other transplant-related modalities that significantly influenced T cell reconstitution in our cohort like donor age and the use of serotherapy. Although the biological background of this finding needs further research, it bears many implications for transplant design. MSD HSCT remains the preferred donor source for many transplant indications as outcomes including GRFS (composite endpoint of graft-versus-host-disease-free and relapse-free survival), relapse-free survival and non-relapse mortality show better results compared to MUD transplantation (23). As Vδ2+ T cells are already known to have important antitumor and anti-infectious capacities, fast reconstitution of these cells might contribute to favorable outcomes in patients with matched related donors.

To what extend immune reconstitution is influenced by the method of stem cell harvest in a T cell replete setting is still unclear. As mentioned above, the study by Minculescu et al. showed superior outcomes in patients with high γδ T cells. Their cohort consisted mainly of adult patients receiving PBSC grafts from MRD/MUD donors and in contrast to our cohort, Vδ2+ T cells were the predominant subset found in the peripheral blood during the first year after transplantation. Nevertheless, they observed a shift towards Vδ1+ subset similar to the distribution in our pediatric cohort during that timeframe (18). If this difference is due to mobilization of peripheral blood stem cells is not clear and further studies are needed to identify the best transplant setting to facilitate fast γδ T cell recovery.

We report a lower cumulative incidence of grade II-IV aGvHD in patients recovering with a high relative abundance of Vδ2+ T cells. At the same time, we observed an inversed Vδ2/Vδ1-ratio after HSCT that has been described by other groups for the first few months after transplantation (22). In our cohort the population inversion persisted until day +240 after HSCT. Patients without extensive aGvHD showed a trend towards normalization of the ratio between day +100 and +240, contrary to patients with higher grade aGvHD. This evidence supports a protective effect of γδ T cells and especially Vδ2+ T cells by sustaining immune homeostasis and thereby avoiding aGvHD in the context of unmanipulated bone marrow transplantation. This hypothesis is supported by findings from pediatric studies with patients receiving TCR αβ/B cell-depleted grafts with high counts of γδ T cells (22). Although patients in this study did not receive GvHD-prophylaxis no severe aGvHD was registered. Still, other reports demonstrated an elevated risk of grade II-IV aGvHD in patients with γδ T cell-enriched graft composition (24) and several studies in mice reported that donor- as well as host-derived γδ T cells can promote aGvHD (25–27). These conflicting results emphasize the need for further research on the role of the different γδ T cell subtypes and the molecular pathways via which γδ T cells affect aGvHD pathogenesis.

Viral infections account for another large part of transplant-associated morbidity and mortality. In our cohort we observed a high EBV infection rate of 51%. One explanation of this finding might be the frequent use of serotherapy, mainly with ATG, in 73% of our patients. ATG is known to delay IR after HSCT and increase the risk of EBV infection and EBV-associated post-transplant lymphoproliferative disorder after HSCT (28).

Liu et al. reported a higher incidence of EBV infection in adult patients recovering with low absolute Vδ2+ T cell counts at day +30 after haploidentical transplantation (29). In our study we could reproduce the same significant association between EBV infection and reduced Vδ2+ counts using the time-averaged ratio R of Vδ2/TCR αβ. Our hypothesis, that early robust Vδ2+ T cell reconstitution might protect patients from developing EBV infection after HSCT is supported by groups that demonstrated the ability of Vδ2+ T cells to recognize and kill EBV-infected cells *in vitro* (29, 30).

Interestingly, we found that absolute Vδ1+ T cell counts were significantly increased at day +100 and +180 in patients with EBV infection. This was accompanied by a reversed Vδ2/Vδ1-ratio in patients with EBV infection between days +60 and +240. Like Vδ2+, Vδ1+ T cells expand upon activation with EBV-infected cells and skewing towards an oligoclonal Vδ1+ T cell repertoire after EBV infection in the context of HSCT has been observed (31). The same study reported that Vδ1+ T cells are capable of lysing EBV infected cells *in vitro*. Taking into consideration that EBV infection in our study occurred at a median of 50 days after transplantation, Vδ1+ expansion in patients with EBV infection after day +60 could be a sign of a targeted immune reaction to clear EBV-infected cells. Furthermore, the high rate of EBV infection can serve as an explanation for the inversed Vδ2/Vδ1-ratio seen in our cohort. Whether Vδ1+ T cells detected in the peripheral blood are mobilized from epithelial tissue – its natural habitat – or proliferate upon contact with EBV-infected cells in a different location is not clear.

While expansion of Vδ1+ T cells after EBV infection is a relatively new finding, several studies have described a possible role of γδ T cells and especially Vδ1+ subset in CMV infection after HSCT (18, 22, 32, 33). Our findings support the existing evidence, adding data from a pediatric study population. We saw a lower cumulative incidence of CMV infection in patients recovering with a high relative abundance of total γδ T cells after HSCT. The Vδ1+ T cell subset expanded between day +60 and day +180 in patients with CMV infection, highlighting its capacity to recognize and kill CMV-infected cells (32). Our study cohort was too small to show any association between CMV infection and relapse rates. In 2011 Elmaagacli et al. reported lower relapse rates in adult AML patients who experienced CMV infection and Scheper et al. provided a possible explanation by showing that non-Vδ2+ T cells are able to cross-recognize leukemia cells and CMV-infected cells *in vitro* (34, 35).

In contrast to EBV and CMV disease we did not find any significant association between the reconstitution of γδ T cell subsets and ADV infection after HSCT. Interestingly, patients with systemic ADV infection had higher rates of grade II-IV aGvHD. This finding is in line with other studies that described aGvHD as a risk factor for ADV infection (36). At the same time, ADV might trigger intestinal aGvHD as hypothesized by a study that found higher rates of intestinal aGvHD in patients that had a positive ADV stool PCR before transplantation (37). Correspondingly, three of four patients in our cohort with intestinal aGvHD also presented with positive ADV PCR in stool and blood.

The results of our study are limited by the single-center design and the heterogeneity of the patient population. Still, it includes a relatively high number of patients for a pediatric HSCT study and all participants uniformly received unmanipulated bone marrow grafts, which is of great importance as graft source has a major impact on immune reconstitution.

In conclusion, we demonstrate that donor choice has a strong influence on γδ T cell reconstitution after pediatric HSCT with unmanipulated bone marrow grafts. Especially Vδ2+ T cell reconstitution was severely hampered in patients with MUD compared to those with MRD, which can contribute to the understanding of favorable outcomes after MRD transplantation. We report a lower cumulative incidence of grade II-IV aGvHD and EBV infection in patients recovering with a high relative abundance of Vδ2+ T cells. This supports a protective role of the Vδ2+ subset early after transplantation, a cell population that bears great potential as part of posttransplant adoptive T cell therapy (38, 39). First studies are currently performed exploring the potential of allogeneic γδ T cell transfer against aGvHD and the relapse of hematological malignancies after HSCT (40). Similar studies using ex vivo expansion and activation of γδ T cells are necessary to evaluate the efficacy in pediatric patients. Due to the high variability of γδ T cell reconstitution dynamics seen early after transplantation the ex vivo approach seems more promising than *in vivo* expansion methods. Establishing an effective but safe cell number for reinfusion will be a crucial point of the analysis.

Additionally, we observed expansion of Vδ1+ T cell subset in patients with CMV as well as with EBV infection. This new association in the context of EBV infection after HSCT can contribute to the process of understanding the role of γδ T cells in anti-virus immunity (41). This opens grounds for future research.

## Data availability statement

The raw data supporting the conclusions of this article will be made available by the authors, without undue reservation.

## Ethics statement

The studies involving humans were approved by The Ethics Committee of Charité – Universitätsmedizin Berlin. The studies were conducted in accordance with the local legislation and institutional requirements. Written informed consent for participation in this study was provided by the participants’ legal guardians/next of kin.

## Author contributions

TM: Visualization, Investigation, Data curation, Writing – original draft. LA: Writing – review & editing, Visualization, Methodology, Formal analysis. FP: Investigation, Data curation, Writing – review & editing. LZ: Formal analysis, Writing – review & editing, Investigation. MS: Methodology, Writing – review & editing. PH: Writing – review & editing, Investigation. AE: Supervision, Writing – review & editing, Investigation. JS: Writing – review & editing, Investigation. AS: Investigation, Writing – review & editing, Supervision. LO: Writing – review & editing, Writing – original draft, Supervision, Project administration, Methodology, Funding acquisition, Conceptualization.
