# Supplementary material for: Lower incidence of grade II-IV acute Graft-versus-Host-Disease in pediatric patients recovering with high Vδ2+ T cells after allogeneic stem cell transplantation with unmanipulated bone marrow grafts: a prospective single-center cohort study
Source: Front Immunol. 2024 Jul 29;15:1433785. doi: 10.3389/fimmu.2024.1433785 (PMC11317287; doi:10.3389/fimmu.2024.1433785)
Supplement: Supplementary file 1 [file DataSheet_1.docx]

**Supplement**

Supplementary Figure 1 | Details on Diagnosis, Donor type, Serotherapy and Conditioning regimens of patients in the study cohort.

Supplementary Figure 2 | **(A)** The separation of the cohort based on high and low Vδ2 population. This is done by maximizing the Jaccard distance in the space of the log relative abundance of Vδ2 and the ranking of the patients based on it. **(B)** The effect size as a function of the cut-off ratio of the time averaged Vδ2 relative abundance Rc. The cut-off is chosen such that the effect size is maximum.


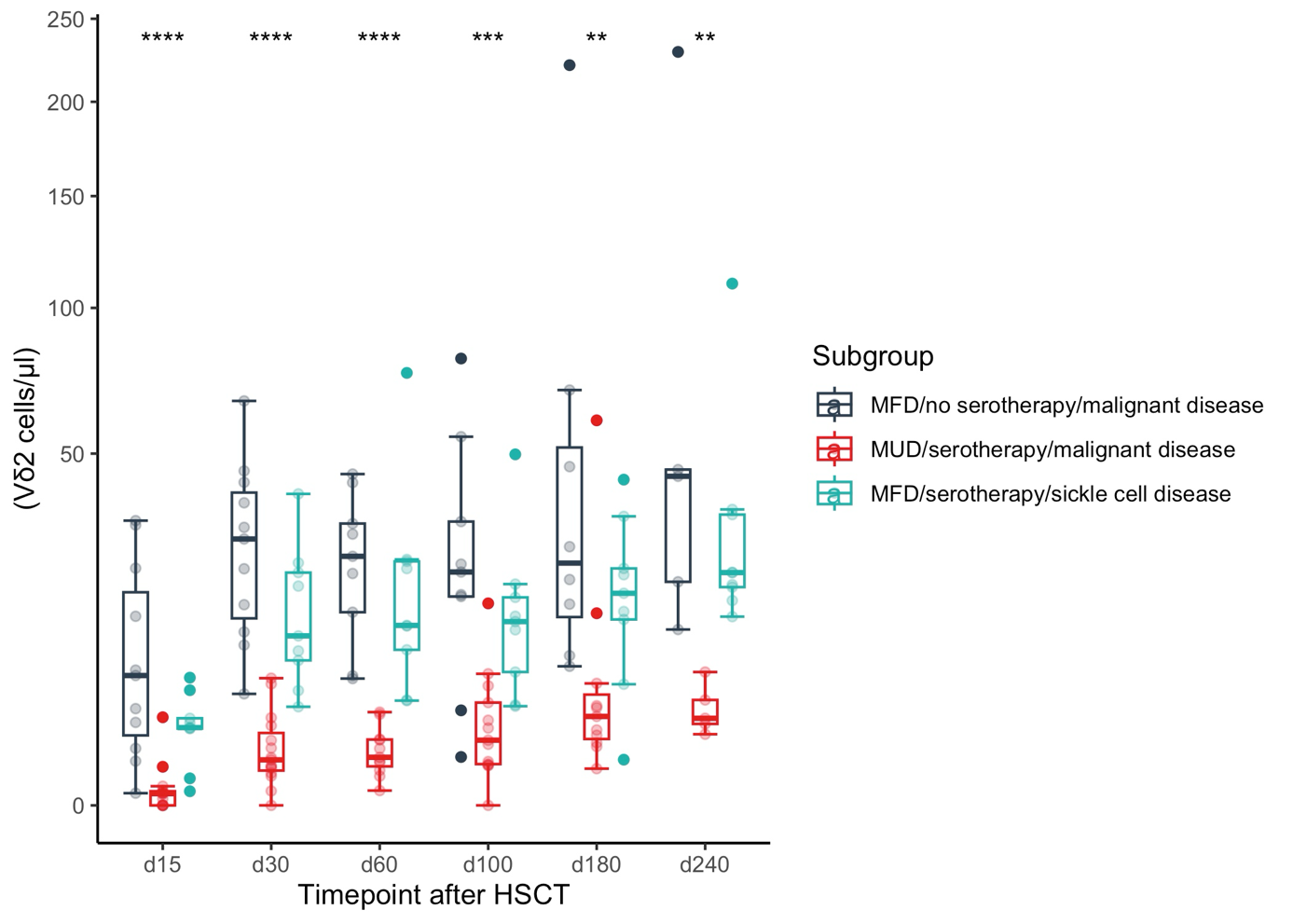


Supplementary Figure 3 | Subgroup analysis of Vδ2+ T cell reconstitution in patients with different combinations of donor type (MRD = matched related donor vs. MUD = matched unrelated donor), serotherapy (yes vs. no) and disease type (malignant disease vs. sickle cell disease). There is no significant difference between patients with malignant disease that received BM from a MRD without previous serotherapy compared to patients with sickle cell disease that received ATG before transplant from a MRD. Significance for multiple group testing (Kruskal-Wallace-Test) is indicated by asterisks.


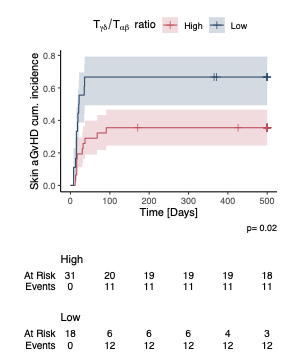


Supplementary Figure 4 | Cumulative incidence of Skin aGvHD in patients with a high vs. low relative abundance of γδ T cells.


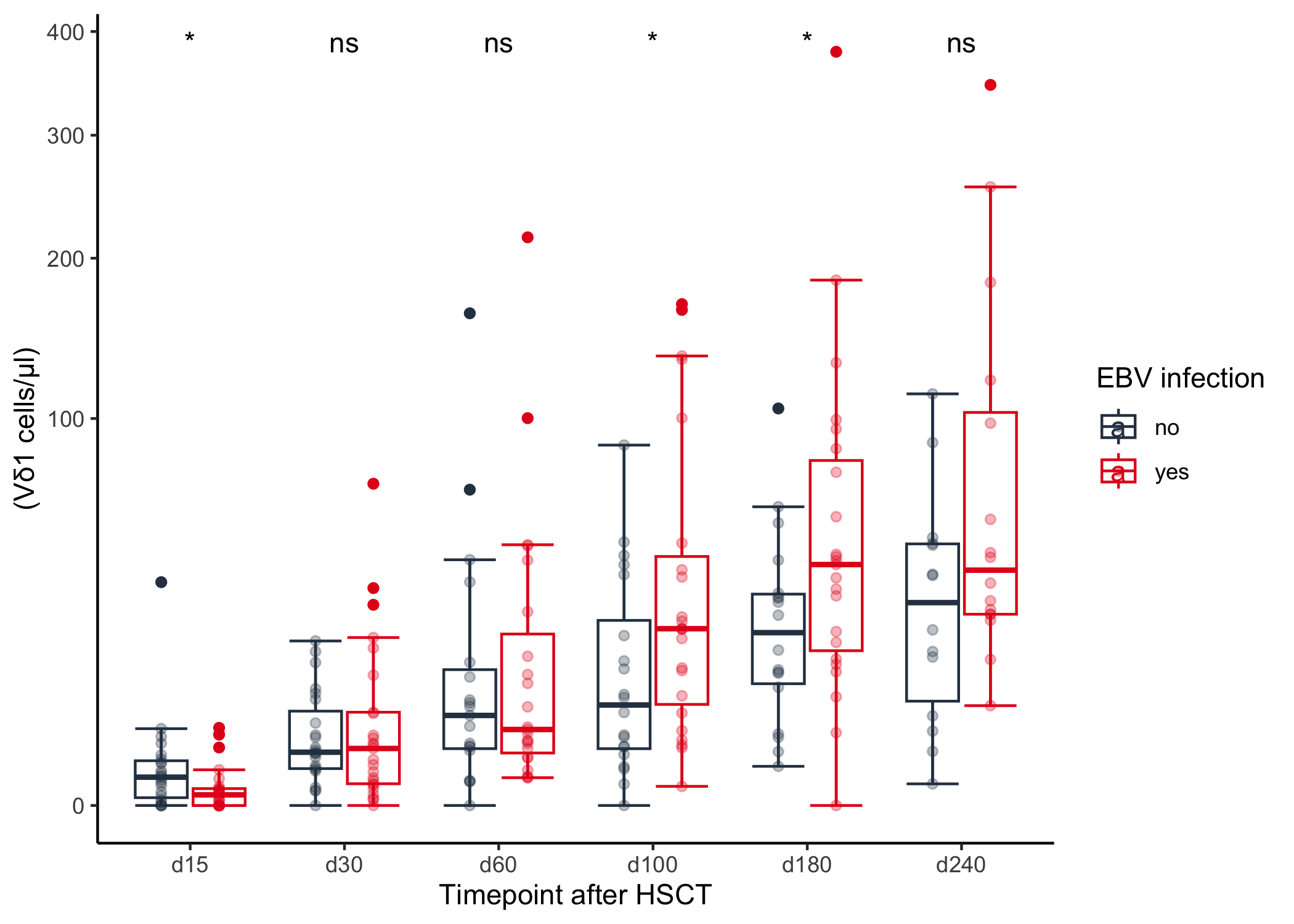


Supplementary Figure 5 | Comparison of absolute Vδ1+ T cell counts in patients with and without EBV infection after transplantation.


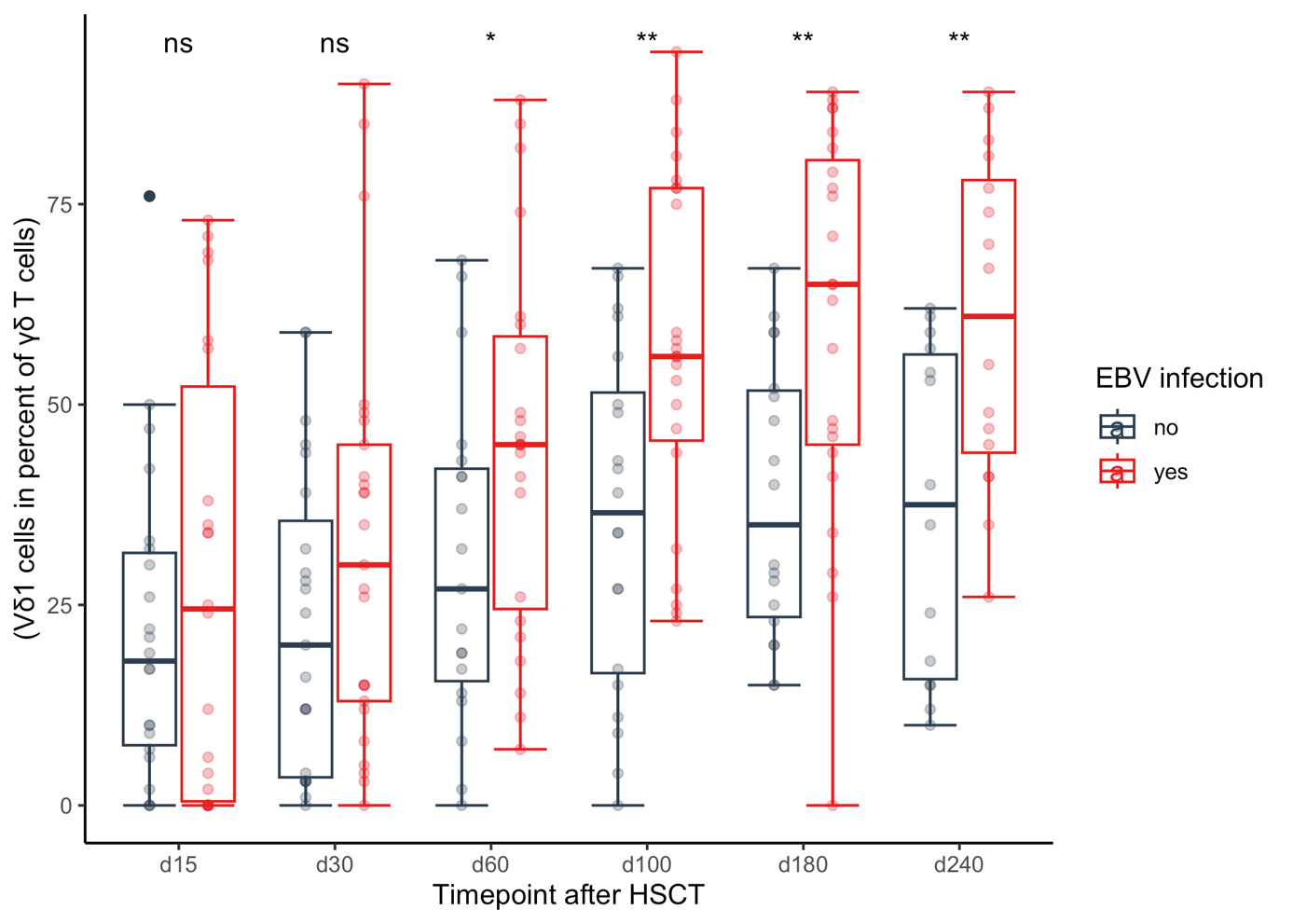


Supplementary Figure 6 | Comparison of Vδ1+ T cells in percent of γδ T cells in patients with and without EBV infection after transplantation.


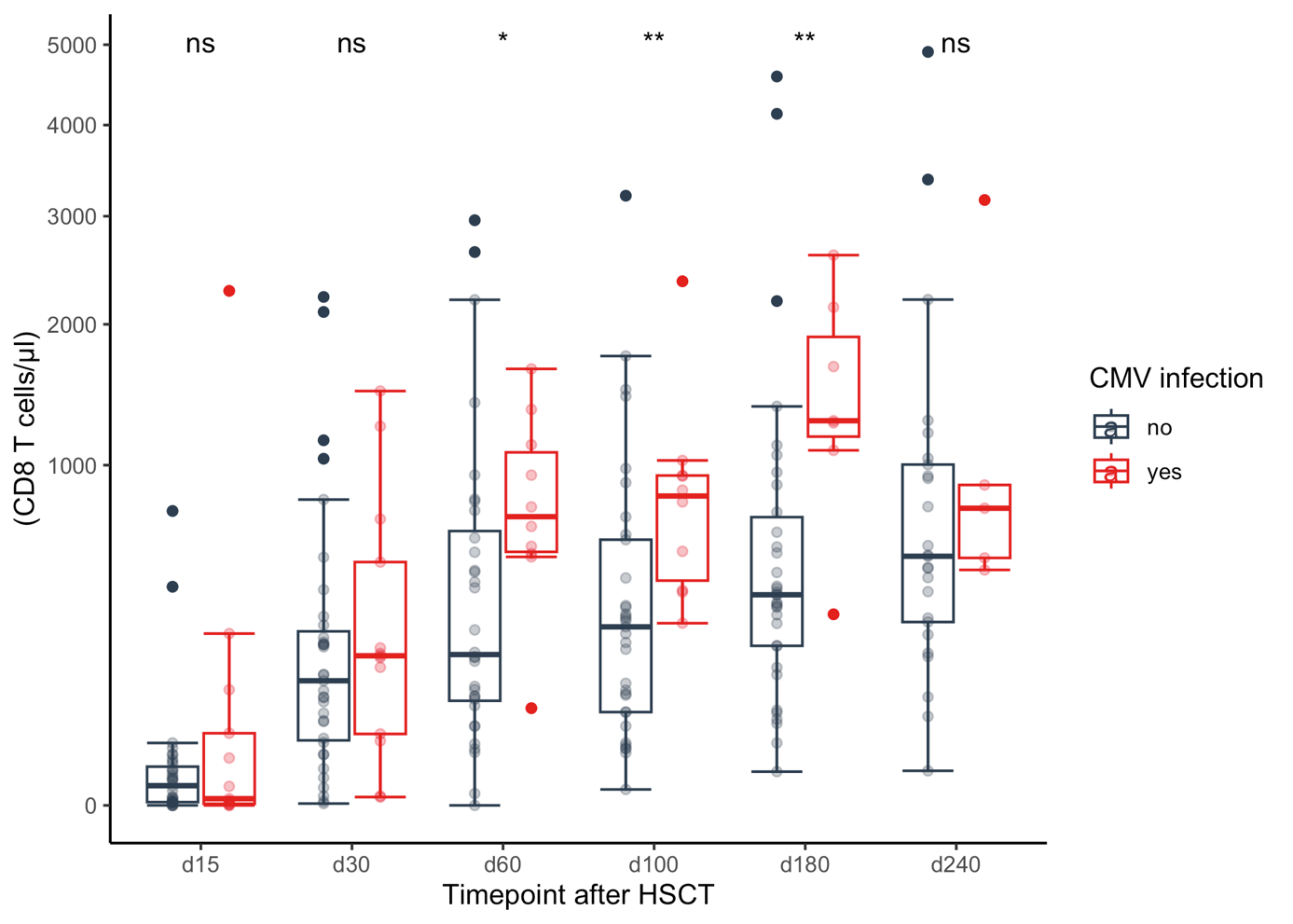


Supplementary Figure 7 | Comparison of absolute CD8 T cell counts in patients with and without CMV infection after transplantation.


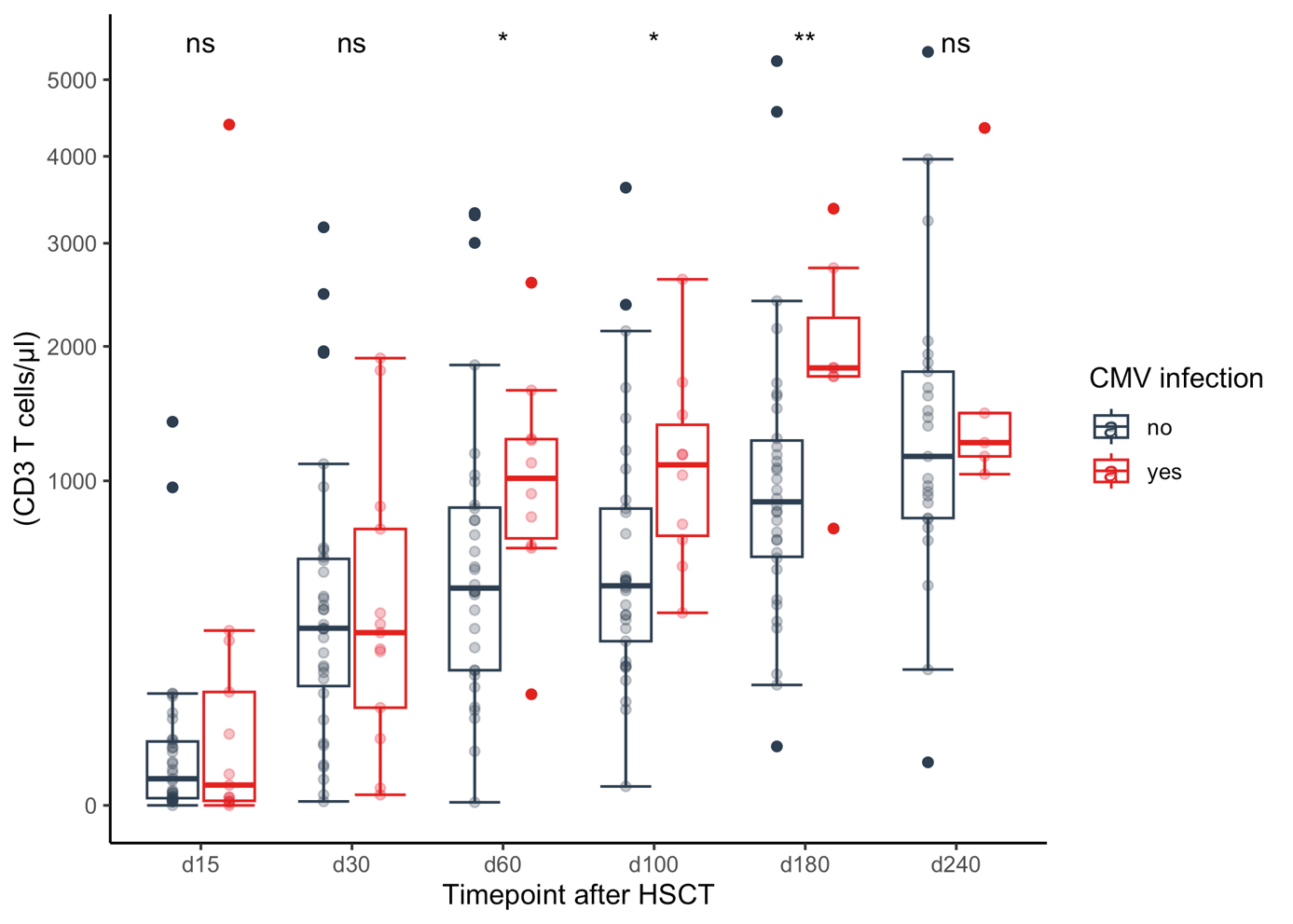


Supplementary Figure 8 | Comparison of absolute CD3 T cell counts in patients with and without CMV infection after transplantation.


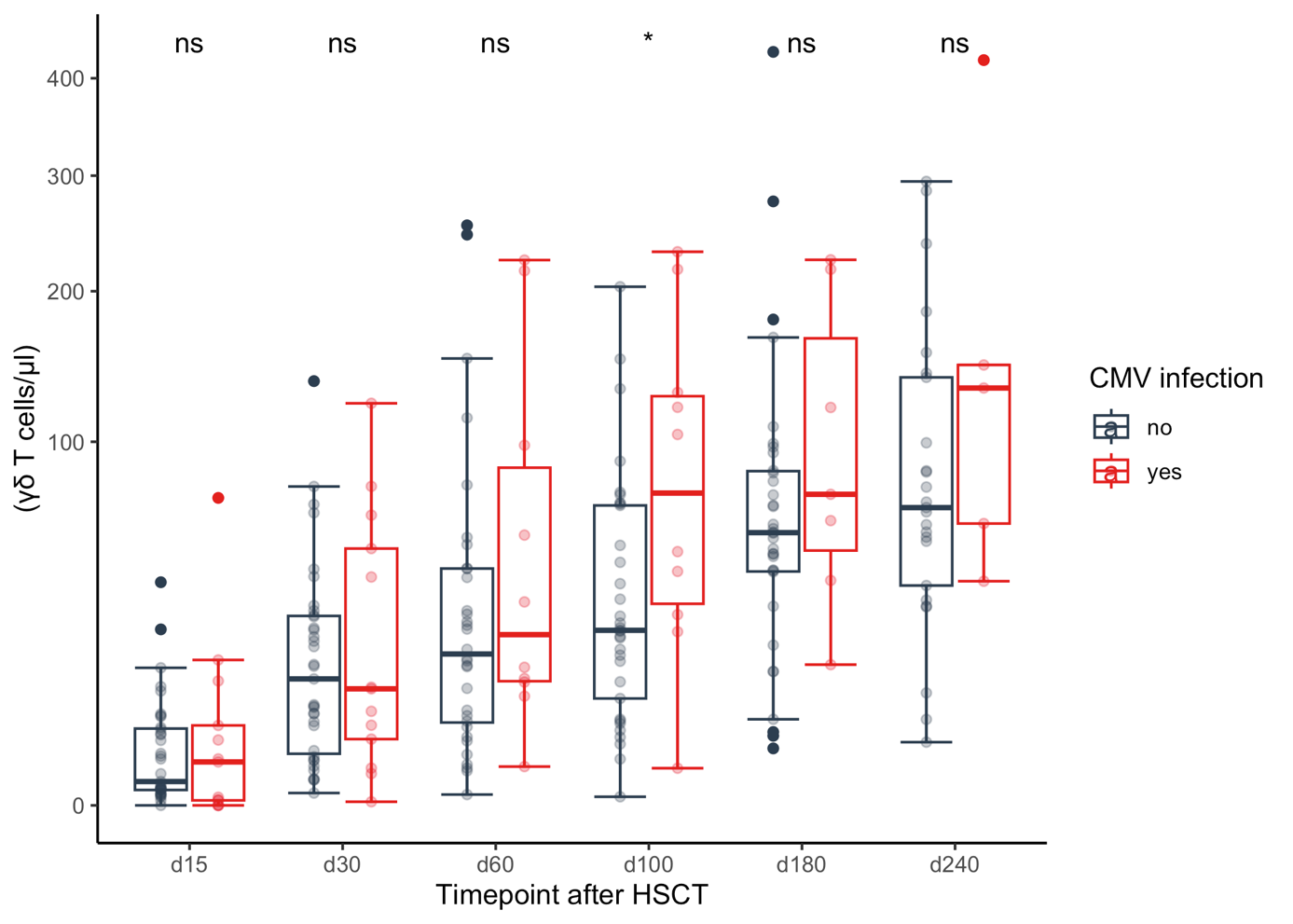


Supplementary Figure 9 | Comparison of absolute γδ T cell counts in patients with and without CMV infection after transplantation.


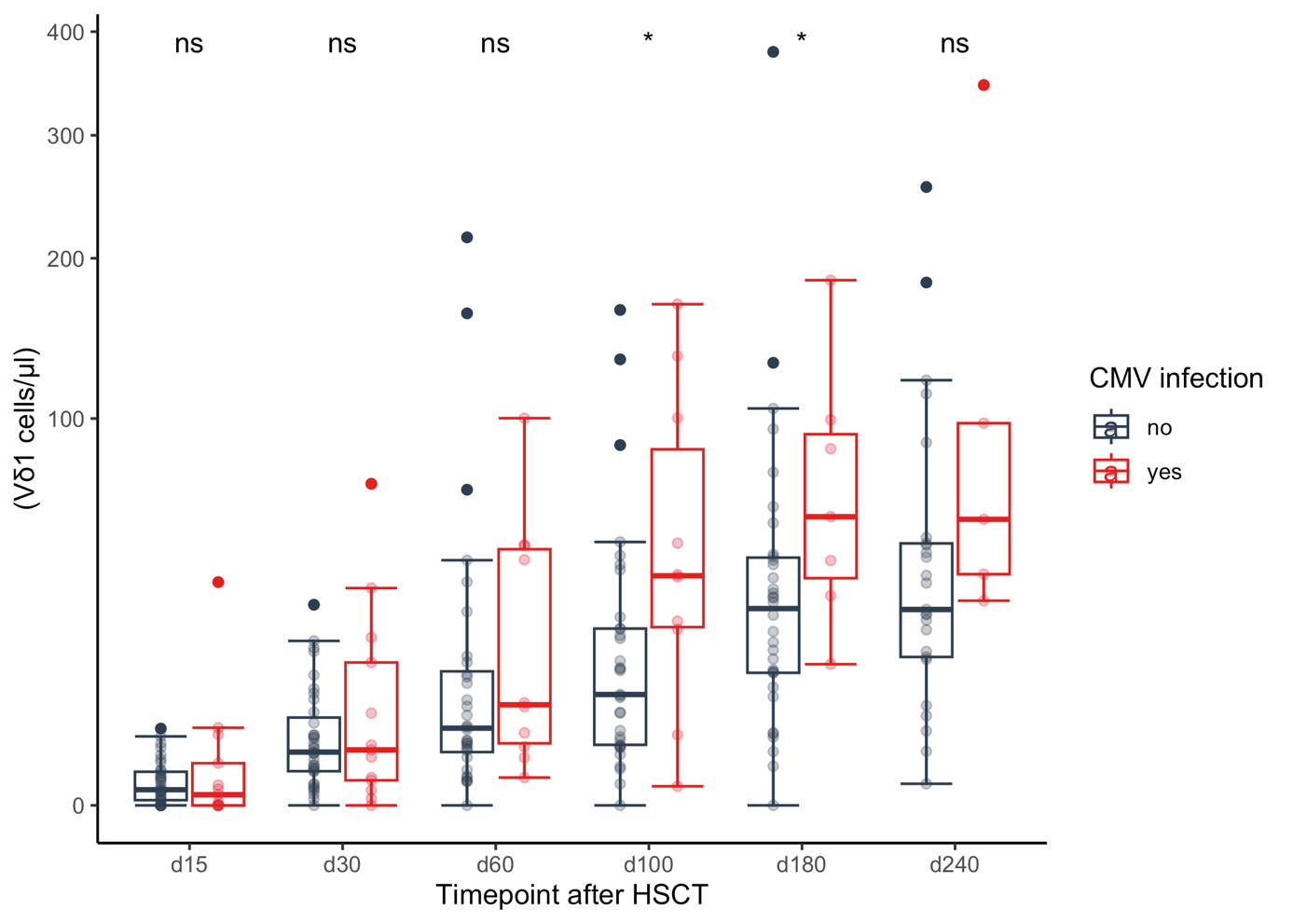


Supplementary Figure 10 | Comparison of absolute Vδ1+ T cell counts in patients with and without CMV infection after transplantation.

Supplementary Figure 11 | Cumulative incidence of CMV reactivation in patients with a high vs. low relative abundance of γδ T cells.
